# *QQS* orphan gene and its interactor *NF‐YC4* reduce susceptibility to pathogens and pests

**DOI:** 10.1111/pbi.12961

**Published:** 2018-07-06

**Authors:** Mingsheng Qi, Wenguang Zheng, Xuefeng Zhao, Jessica D. Hohenstein, Yuba Kandel, Seth O'Conner, Yifan Wang, Chuanlong Du, Dan Nettleton, Gustavo C. MacIntosh, Gregory L. Tylka, Eve S. Wurtele, Steven A. Whitham, Ling Li

**Affiliations:** ^1^ Department of Plant Pathology and Microbiology Iowa State University Ames IA USA; ^2^ Department of Genetics, Development and Cell Biology Iowa State University Ames IA USA; ^3^ Laurence H. Baker Center for Bioinformatics and Biological Statistics Iowa State University Ames IA USA; ^4^ Roy J. Carver Department of Biochemistry, Biophysics and Molecular Biology Iowa State University Ames IA USA; ^5^ Department of Biological Sciences Mississippi State University Starkville MS USA; ^6^ Department of Statistics Iowa State University Ames IA USA; ^7^ Center for Metabolic Biology Iowa State University Ames IA USA

**Keywords:** carbon and nitrogen partitioning, *NF‐YC4*, pathogen, pest, orphan, *QQS*

## Abstract

Enhancing the nutritional quality and disease resistance of crops without sacrificing productivity is a key issue for developing varieties that are valuable to farmers and for simultaneously improving food security and sustainability. Expression of the *Arabidopsis thaliana* species‐specific *AtQQS* (*Qua‐Quine Starch*) orphan gene or its interactor, NF‐YC4 (Nuclear Factor Y, subunit C4), has been shown to increase levels of leaf/seed protein without affecting the growth and yield of agronomic species. Here, we demonstrate that overexpression of *AtQQS* and *NF‐YC4* in Arabidopsis and soybean enhances resistance/reduces susceptibility to viruses, bacteria, fungi, aphids and soybean cyst nematodes. A series of Arabidopsis mutants in starch metabolism were used to explore the relationships between *QQS* expression, carbon and nitrogen partitioning, and defense. The enhanced basal defenses mediated by QQS were independent of changes in protein/carbohydrate composition of the plants. We demonstrate that either *AtQQS* or *NF‐YC4* overexpression in Arabidopsis and in soybean reduces susceptibility of these plants to pathogens/pests. Transgenic soybean lines overexpressing *NF‐YC4* produce seeds with increased protein while maintaining healthy growth. Pull‐down studies reveal that QQS interacts with human NF‐YC, as well as with Arabidopsis NF‐YC4, and indicate two QQS binding sites near the NF‐YC‐histone‐binding domain. A new model for QQS interaction with NF‐YC is speculated. Our findings illustrate the potential of QQS and NF‐YC4 to increase protein and improve defensive traits in crops, overcoming the normal growth‐defense trade‐offs.

## Introduction

Crop plants grow in dynamic environments that abound with challenges. Plants have evolved highly sophisticated immune systems to detect and defend themselves from pathogens/pests (Chisholm *et al*., 2006; Jones and Dangl, 2006), yet, most economically important crops incur significant yield losses due to diseases (Wulff *et al*., 2011). Genetic resistance is ideal as an approach to combat disease because it naturally protects crops without additional chemical or mechanical inputs by farmers. To protect crops from a devastating disease, resistant lines are often developed that utilize single genes conferring high levels of resistance to the specific pest or pathogen (Wulff *et al*., 2011).

Broad‐spectrum resistance to multiple pathogens would be an extremely valuable trait. It has been achieved by inducing constitutively active defense responses; however, maintaining constitutive defenses is often energetically costly, impairing plant growth and yield (Bolton, 2009; Heil *et al*., 2000). For example, after infection by avirulent isolates of powdery mildew that trigger immune responses, seeds of barley have decreased weight and protein content (Smedegaard‐Petersen and Stolen, 1981). Also, in addition to being resistant to a variety of pathogens, Arabidopsis mutants with constitutively active immunity such as the *defense no death* (*dnd1*) and *constitutive PR gene expression* (*cpr*) lines display a dwarfed morphology (Bowling *et al*., 1997; Clarke, 1998; Clough *et al*., 2000; Genger *et al*., 2008). Silencing the expression of *MAP kinase 4* (*MAPK4*) in soybean, which constitutively activates salicylic acid (SA)‐based defenses, results in plants that develop spontaneous necrosis and are severely stunted, in addition to being more resistant to pathogens (Liu *et al*., 2011). In contrast, mutants with suppressed immune systems exhibit increased fitness under pathogen‐free conditions, growing taller and producing more seeds than wild‐type (WT) plants (Heil and Baldwin, 2002). This ‘growth‐defense trade‐off’ phenomenon is the current paradigm, and novel strategies are needed to maximize crop fitness while enhancing broad‐spectrum defense. Genes that limit the growth of diverse plant pathogens/pests without impairing crop growth and yield could provide new traits for breeding resilient crops (Huot *et al*., 2014).

Each sequenced species, prokaryote or eukaryote, contains protein‐coding genes that are unique to that particular species (orphan genes) (Arendsee *et al*., 2014; Carvunis *et al*., 2012; Gollery *et al*., 2006; Wissler *et al*., 2013). Little is known about the functional significance of the vast majority of orphan genes (Arendsee *et al*., 2014). *Qua Quine Starch* (*QQS*, At3g30720) is an *Arabidopsis thaliana* orphan gene, encoding a small protein of only 59 aa with no known functional/structural motifs. It has no homology with proteins of genes of other species, including those of *A. lyrata* and *A. halerieii* (Li *et al*., 2009, 2015). *QQS* regulates carbon and nitrogen partitioning to starch and protein in Arabidopsis, and also in the leaves and seeds of transgenic *QQS*‐expressing soybean, corn and rice (Li and Wurtele, 2015b; Li *et al*., 2009, 2015). Transgenic plants with perturbed *QQS* expression have altered starch and protein accumulation but normal development and morphology. Overexpression of *QQS* increases protein content and decreases starch content, while down‐regulation of *QQS* decreases protein and increases starch (Li and Wurtele, 2012, 2015a,b; Li *et al*., 2009, 2015).

QQS interacts with the Nuclear Factor Y subunit C4 protein (NF‐YC4; At5g63470) (Li *et al*., 2015), a subunit of the heterotrimeric NF‐YA/NF‐YB/NF‐YC transcription factor, which is conserved across eukaryotes (Laloum *et al*., 2013; Nardini *et al*., 2013). NF‐Ys comprise a large family in plant species, with a total of 30 NF‐Ys (10 NF‐YCs) in Arabidopsis (Laloum *et al*., 2013; Li *et al*., 2015; Petroni *et al*., 2012). Although AtNF‐YC4‐QQS interaction has been demonstrated by several methods *in vitro* and *in vivo* (Li *et al*., 2015)*,* it has not been ruled out that QQS does not interact with other plant NF‐YCs, or indeed other plant molecules. However, overexpression of *AtNF‐YC4* in Arabidopsis mimics the *QQS*‐overexpression phenotype, increasing protein and decreasing starch (Li *et al*., 2015). Similar to the consequence of *QQS* overexpression, the overexpression of *NF‐YC4* does not impact morphology/yield (Li and Wurtele, 2015b; Li *et al*., 2009, 2015).

One major function of orphan genes may be to increase the survival of organisms in new environments (Arendsee *et al*., 2014; Carvunis *et al*., 2012; Lacombe *et al*., 2010; Li *et al*., 2009; Luhua *et al*., 2013; Wissler *et al*., 2013). Reflective of this general concept about orphan genes and because the level of *QQS* mRNA is responsive to multiple abiotic and biotic stresses (Arendsee *et al*., 2014; Li and Wurtele, 2015b; Li *et al*., 2009), we postulated that *QQS* might play a role in plant responses to pathogens/pests, in addition to its established role in carbohydrate and protein composition. A function for *QQS* in plant defense would be consistent with our hypothesis that this orphan gene improved Arabidopsis' ability to adapt to changing biotic stresses (Arendsee *et al*., 2014).

To determine whether *QQS* and *NF‐YC4* might function in plant defense, we examined existing and new transcriptomic data from Arabidopsis subjected to varying biotic conditions or with perturbed *QQS* levels, identifying that expression of *QQS* and *NF‐YC4* is altered in response to biotic stresses, and changes in *QQS* expression can alter expression of genes involved in plant responses to pathogens/herbivores/abiotic stresses. These results led us to evaluate the susceptibility to pathogens/pests of Arabidopsis lines that had overexpression/down‐regulation/loss‐of‐function of *QQS* or *NF‐YC4* (Li and Wurtele, 2015b; Li *et al*., 2009, 2015), and mutants with different combinations of levels of starch accumulation and expression of *QQS* (Delvalle *et al*., 2005; Lu *et al*., 2008; Wattebled *et al*., 2008; Zhang *et al*., 2005). We further determined whether soybean lines that express *QQS* (Li and Wurtele, 2015b; Li *et al*., 2015) or overexpress the soybean homolog of *NF‐YC4* also had altered susceptibility. The resultant data show that expression of *QQS* or overexpression of its interacting partner *NF‐YC4* can confer broad‐spectrum defense while maintaining normal growth. Further, we show that an interaction between QQS and NF‐YCs can extend to a protein as evolutionarily divergent as human NF‐YC and explore the segment of QQS associated with this interaction. We used a computational modelling approach and proposed binding sites for QQS peptides at the N‐terminus of NF‐YC near the histone‐binding domain. We confirmed these binding sites experimentally and speculated a model that QQS interacts with NF‐YC.

Our results demonstrate that it is possible to simultaneously enhance protein content and defense responses without impairing plant growth; this is true for *A. thaliana*, the species that naturally contains *QQS*, and for soybean, an important crop plant that has no *QQS* homolog. This ability of QQS and NF‐YC4 to regulate the allocation of carbon and nitrogen as well as promote plant defense indicates that it may participate in a regulatory hub that bypasses the trade‐off between plant growth and defense.

## Results

### *QQS* and *NF‐YC4* mRNA expressions are altered in response to biotic stresses

To examine the expression of *QQS* and *NF‐YC4* in response to pathogens, we analysed published transcriptomic data sets in which Arabidopsis plants had been inoculated with a virus, bacterium or oomycete. At 120 h after inoculation (HAI) with TuMV‐GFP (*Turnip mosaic virus* expressing GFP) (Yang *et al*., 2007), the *QQS* transcript level was significantly decreased in tissues near the centre of fluorescent GFP foci (zones 0 and 1) (*P *=* *0.01, 0.04) in which TuMV‐GFP had the most accumulation (Figure S1a). No significant changes were detected for the *NF*‐*YC4* transcript; however, *NF*‐*YC4* expression was near background levels in these samples, making it difficult to determine whether TuMV‐GFP affected its expression. Arabidopsis Col‐0 plants are susceptible to the bacterial pathogen *Pseudomonas syringae* pv. *tomato* (*Pst*) strain DC3000 (Thilmony *et al*., 2006). *QQS* transcript level was significantly reduced in plants infected with *Pst* DC3000 at 7 (*P *=* *0.001) and 24 HAI (*P *=* *0.01), while *NF‐YC4* transcript level was not significantly affected, although they trend downward at both time points (Figure S1b). In an oomycete (*Phytophthora infestans*, causing a nonhost resistance response in Arabidopsis) infection experiment (Figure S1c), there were no significant differences in *QQS* expression between inoculated and mock‐inoculated plants, although there appeared to be a trend in which *QQS* was initially down‐regulated at 6 HAI but not at later time points (12 and 24 HAI). *NF‐YC4* was initially down‐regulated at 6 HAI (*P *<* *0.001), but by 24 HAI, its mRNA expression was similar in inoculated and mock‐inoculated plants. Taken together, the altered expression of *QQS*, and to a lesser extent *NF‐YC4*, in response to diverse pathogens is consistent with a model in which *QQS* plays a role in plant–microbe interactions. Based on these observations, we hypothesized that *QQS* and *NF‐YC4* may regulate the expression of defense genes and/or the susceptibility of Arabidopsis to pathogens and possibly pests.

To determine if and how *QQS* affects expression of defense and other genes under control conditions, RNA‐Seq (sequencing) was performed on *AtQQS* RNAi (RNA interference), *AtQQS‐OE* (overexpressing) and WT Col‐0* Arabidopsis plants grown under unstressed conditions (Li *et al*., 2015). Six hundred and five genes were differentially expressed in *AtQQS‐OE* plants at a false discovery rate (FDR) ≤0.05 (Table S1a). In contrast, no gene was differentially expressed in the *AtQQS* RNAi plants at this FDR cut‐off (Table S1b). Among the differentially expressed genes in *AtQQS‐OE* plants, callose biosynthesis, l‐*N* ^δ^‐acetylornithine biosynthesis and monoterpene biosynthesis pathways were overrepresented (Li *et al*., 2015) (Table S1c). These pathways are associated with plant responses to pathogens/herbivores/abiotic stresses (Chen and Kim, 2009; Lewinsohn *et al*., 1991; Serrano *et al*., 2014; Shah, 2003).

To further explore the potential relationship between *QQS* and defense response genes, we selected 13 genes considered as markers for different plant defense responses (Chico *et al*., 2014; Onkokesung *et al*., 2014; Seo *et al*., 2001; Song *et al*., 2014; Truman *et al*., 2006; Zhang *et al*., 2014) and examined the expression of these genes in the RNA‐Seq data set using a *P *<* *0.05 cut‐off (Table S2). This analysis identified only four genes that were significantly induced and one that was significantly down‐regulated. These modest effects of *QQS‐OE* or *QQS* RNAi on the expression of canonical plant immune response genes led us to conclude that *QQS* overexpression does not induce a constitutive large‐scale activation of plant defense responses in the absence of biotic stress.

### *QQS* and *NF‐YC4* enhance plant antiviral and antibacterial immune responses

**Figure 1:**
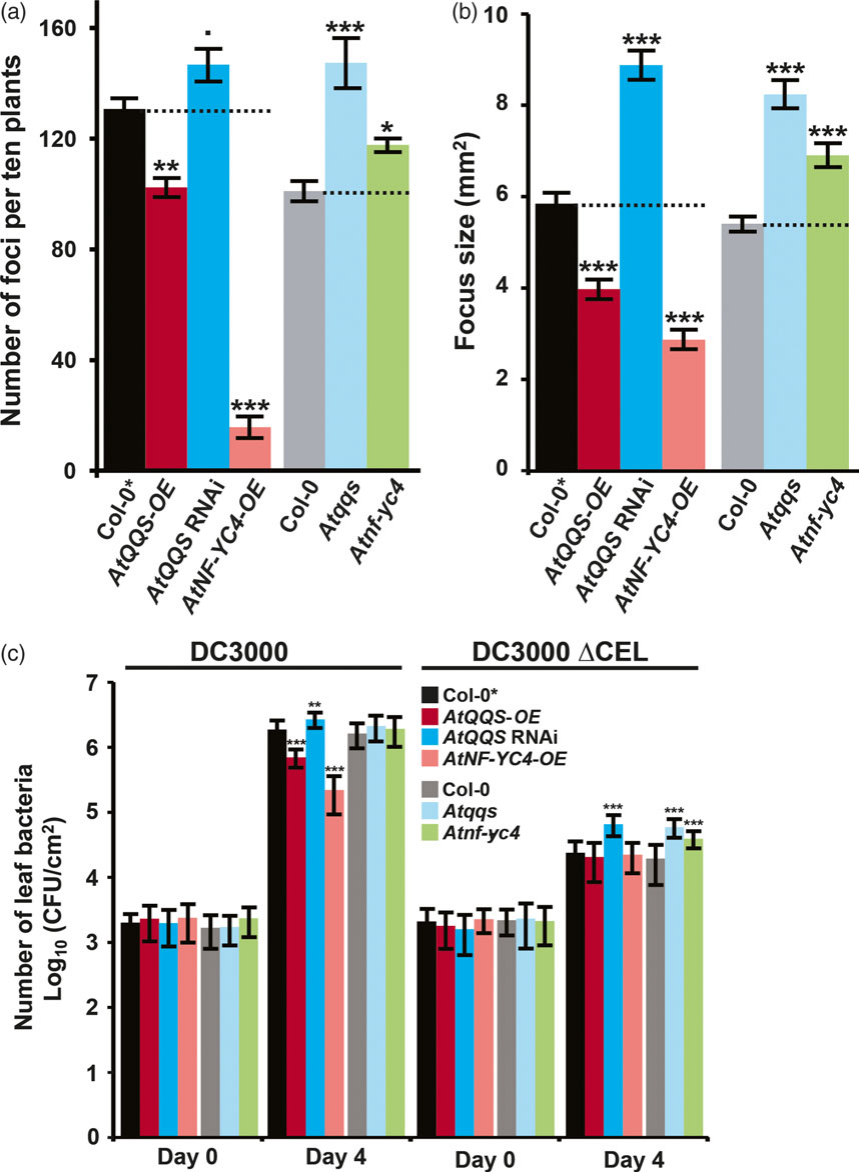
Arabidopsis mutants of QQS and its interactor NF‐YC4 have altered susceptibility to viral and bacterial infection. Mutants: transgenic *AtQQS* RNAi, *AtQQS‐OE* and *AtNF‐YC4‐OE* in Arabidopsis Col‐0* (few trichomes), and T‐DNA knockout mutants *Atqqs*, and *Atnf‐yc4* in Col‐0 (trichomes). (a) The numbers of TuMV‐GFP infection foci at 120 HAI, (b) the sizes of TuMV‐GFP infection foci at 120 HAI and (c) the growth of the *Pst* DC3000 (DC3000) and CUCPB5115 (*Pst* DC3000 ΔCEL) bacterial strains were altered in the mutants. CFU, colony forming units. All data in bar charts show mean ± SE (standard error), *n* = 3. Statistical significance was determined as described in Appendix S1 ‘Experiment design and statistical methods’: ***P <* *0.001; **P <* *0.01; *P <* *0.05; •*P *<* *0.1.

To test the hypothesis that QQS and its interactor NF‐YC4 affect Arabidopsis susceptibility to pathogens/pests, we tested responses to infection with representative pathogens (virus and bacteria), and we also sought to determine whether these responses could be extended to pests (aphids and plant–parasitic nematodes). TuMV‐GFP was inoculated on Arabidopsis lines with genetically induced differences in *QQS* or *NF‐YC4* expression: transgenic *AtQQS* RNAi, *AtQQS*‐*OE* and *AtNF‐YC4‐OE* in Col‐0* (few trichomes), and T‐DNA knockout mutants *Atqqs*, and *Atnf‐yc4* in Col‐0 (trichomes). Only one representative line per genotype was used due to space limitations. At 5 days post inoculation (DPI), the numbers of TuMV‐GFP foci were reduced by 22% in *AtQQS‐OE* plants compared with the controls (*P *=* *0.001), while they were increased by 12% (*P *=* *0.096) and 46% (*P *<* *0.001) in *AtQQS* RNAi and *Atqqs* plants (Figure 1a). Similarly, *AtNF‐YC4‐OE* had an 88% decrease (*P *<* *0.001), and *Atnf‐yc4* plants had a 17% increase (*P *<* *0.05). The sizes of foci followed a similar trend to the numbers of foci (Figure 1b): *Atqqs*,* AtQQS* RNAi and *Atnf‐yc4* plants had larger foci by 28%, 53% and 52% (*P *<* *0.001 for all). In contrast, foci on *AtQQS‐OE* and *AtNF‐YC4‐OE* plants were 32% and 51% smaller (*P *<* *0.001 for both). Thus, overexpressing either *AtQQS* or *AtNF‐YC4* impairs viral infection, while silencing or knocking out these genes enhances viral infection.

To test the effects of *QQS* and *NF‐YC4* expression on the growth of a bacterial pathogen, we inoculated the *QQS* and *NF‐YC4* mutants with *Pst* DC3000 or the nonvirulent *Pst* DC3000 ΔCEL mutant (CUCPB5115). *Pst* DC3000 ΔCEL lacks multiple effector genes and grows poorly *in planta* because it cannot suppress basal defenses (Badel *et al*., 2006). As expected, *Pst* DC3000 grew ~1000‐fold, whereas ΔCEL grew ~10‐fold in WT plants by 4 DPI (Figure 1c). However, the growth of *Pst* DC3000 was decreased by 63% in *AtQQS‐OE* (*P *<* *0.01) and 88% in *AtNF‐YC4‐OE* (*P *<* *0.001), and not significantly altered in the *AtQQS* RNAi, *Atqqs* or *Atnf‐yc4*, when compared to WT plants.

The growth of *Pst* DC3000 ΔCEL was not significantly different in *AtQQS‐OE*,* AtNF‐YC4‐OE* and WT plants. However, its growth was significantly increased in *AtQQS* RNAi, *Atqqs* or *Atnf‐yc4* plants (174%, 207% and 102% increase, *P *=* *0.001, <0.001, <0.01) (Figure 1c). Overall, these altered growth patterns indicate that overexpressing either *AtQQS* or *AtNF‐YC4* enhances plant immunity to a robust bacterial pathogen. In contrast, silencing or knocking out *AtQQS* or *AtNF‐YC4* impairs plant immunity, enabling the nonvirulent ΔCEL to grow better. Combined with the TuMV‐GFP results, these data indicate that *AtQQS* and *AtNF‐YC4* positively regulate plant immunity to these bacterial and viral strains.

### Expression of *QQS* and overexpression of *NF‐YC4* in transgenic soybeans enhances resistance to viral and bacterial pathogens

**Figure 2:**
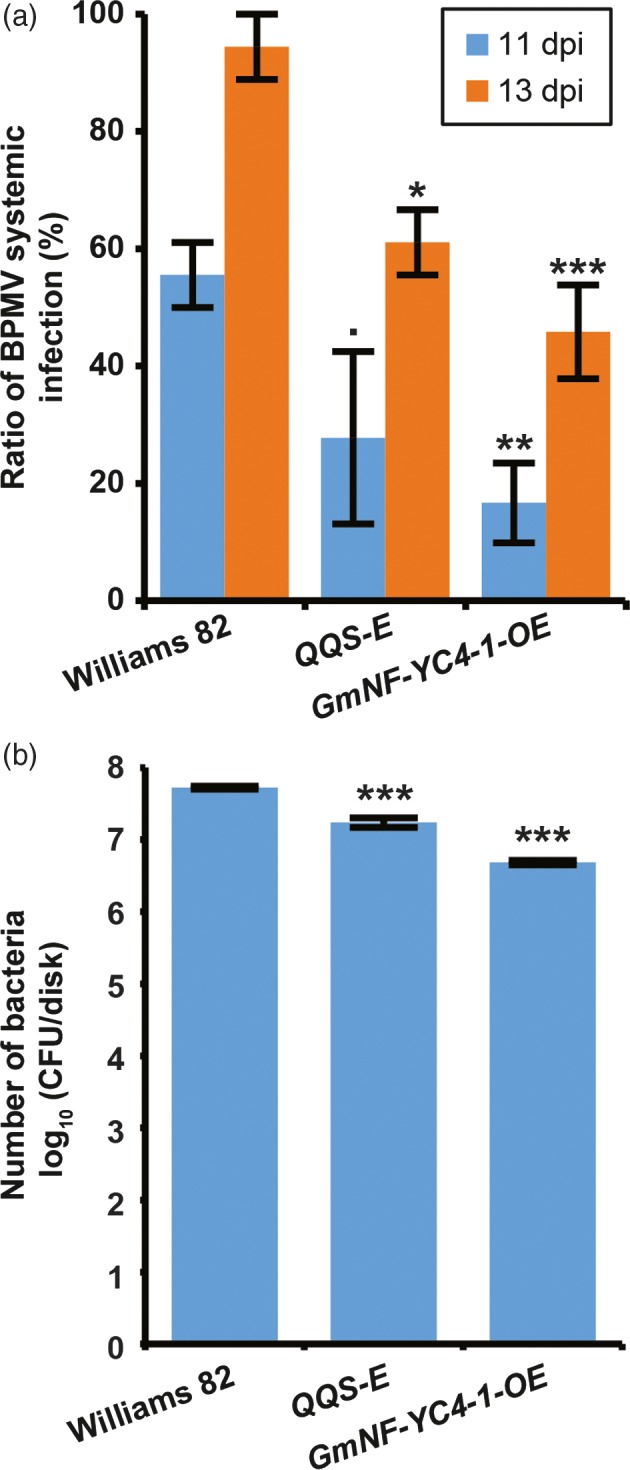
Transgenic soybean lines constitutively expressing *AtQQS* or overexpressing *GmNF‐YC4‐1* had decreased viral and bacterial infection. (a) Systemic infection of BPMV was decreased at both 11 and 13 DPI. (b) Growth of *Psg*R4 was decreased at 7 DPI. CFU, colony forming units. All data in bar charts show mean ± SE,* n *=* *3. ***P <* *0.001; **P <* *0.01; *P <* *0.05; •*P *<* *0.1.

To test if QQS and NF‐YC4 could affect soybean‐pathogen interactions, transgenic soybean lines expressing *AtQQS* (*AtQQS‐E*) or overexpressing *GmNF‐YC4‐1* (*GmNF‐YC4‐1‐OE*) were inoculated with *Bean pod mottle virus* (BPMV), and *P. syringae* pv. *glycinea* Race 4 (*Psg*R4) (causing the disease bacterial blight). Systemic infection of BPMV was decreased in transgenic soybean at both 11 (*P *=* *0.084, 0.008 for *AtQQS‐E* and *GmNF‐YC4‐1‐OE*, respectively) and 13 DPI (*P *=* *0.035, 0.004) (Figure 2a). Growth of *Psg*R4 was decreased by 55% and 62% in *AtQQS‐E* and *GmNF‐YC4‐1‐OE* compared to Williams 82 control plants (*P *=* *0.0003, 0.00002) (Figure 2b). These data show that AtQQS and GmNF‐YC4‐1 can enhance soybean immunity similar to our observations from Arabidopsis.

### Decreased susceptibility to aphids in Arabidopsis and soybean plants overexpressing *QQS* and *NF‐YC4*

To determine whether QQS and NF‐YC4 might also enhance defense against aphids, the Arabidopsis *QQS* and *NF‐YC4* mutants were infested with green peach aphids (*Myzus persicae*). Green peach aphid population growth was compromised by 27% in the *AtQQS‐OE* compared with the controls (*P *=* *0.04), and 10% (*P *=* *0.34) in the *NF‐YC4‐OE* plants. Aphid population growth was increased by 3%, 16% and 28% in the *AtQQS* RNAi, *Atqqs* and *Atnf‐yc4* knockout mutants; however, these differences were not statistically significant (*P *=* *0.86, 0.36, 0.12) (Figure S2). Overall, overexpression of *AtQQS* seems to increase resistance to pests such as aphids in Arabidopsis.

**Figure 3:**
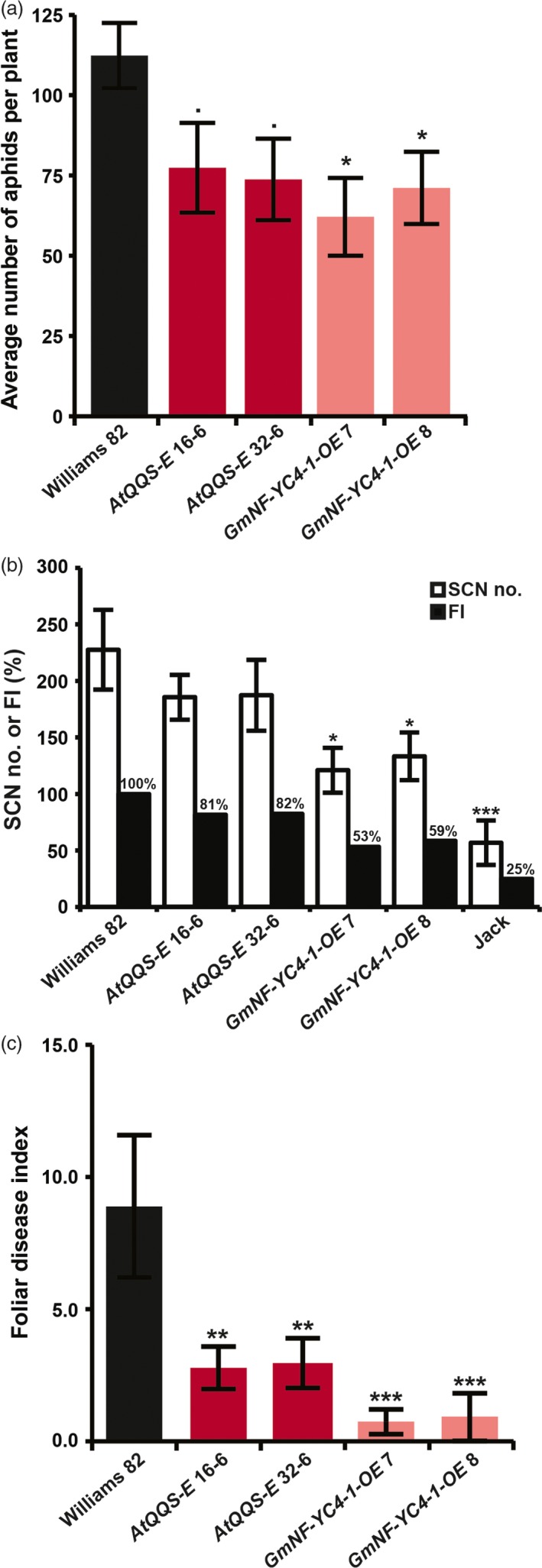
Aphid, SCN and SDS performance were altered in soybean *AtQQS‐E* and *GmNF‐YC4‐1‐OE* mutants. (a) Aphid number, (b) number of soybean cyst nematode females and (c) foliar disease index were decreased in mutants. FI, female index; Williams 82 and Jack lines of soybean, controls for highly susceptible and resistant, respectively. Bar graphs show mean ± SE,* n *=* *10 (a) or 6 (b and c). ***P <* *0.001; **P <* *0.01; *P <* *0.05; •*P *<* *0.1.

To test whether soybean lines expressing *QQS* or overexpressing *NF‐YC4* have a similar anti‐aphid phenotype, the *AtQQS‐E*,* GmNF‐YC4‐1‐OE* and control soybean lines were infested with soybean aphids (*Aphis glycines*). Soybean aphid population growth was compromised in *AtQQS‐E* by 31%–34% (*P *=* *0.09, 0.06) and in *GmNF‐YC4‐1‐OE* lines by 37%–45% (*P *=* *0.02, 0.05) (Figure 3a), demonstrating that *AtQQS* and *GmNF‐YC4* mediate reduced susceptibility to aphids.

### Decreased susceptibility to nematodes in soybean plants expressing *QQS* or overexpressing *NF‐YC4*

The soybean cyst nematode (SCN, *Heterodera glycines*) infects soybean and causes significant yield losses (Niblack *et al*., 2002). To investigate whether *AtQQS* or *GmNF‐YC4* could enhance nematode defense in soybean, the roots of *AtQQS‐E* and *GmNF‐YC4‐1‐OE,* and control soybean lines were grown in soil infested with SCN. The number of SCN females on the roots after a single generation was decreased in the *GmNF‐YC4‐1‐OE* lines by 41%–47% (*P *=* *0.04, 0.05), with a similar, although not significant, trend observed for the *AtQQS‐E* soybean lines (reductions of 18%–19%; *P *=* *0.85, 0.28) (Figures 3b and S3).

### Decreased sudden death syndrome symptoms in soybean plants expressing *QQS* or overexpressing *NF‐YC4* in the field

*AtQQS‐E, GmNF‐YC4‐1‐OE* and WT soybean plants were grown in the field, inoculated with *Fusarium virguliforme*, the fungus that causes sudden death syndrome (SDS). Foliar SDS symptoms started to appear at growth stage R5 (beginning of pod filling). There was a significant difference in foliar disease index (FDX = disease incidence × disease severity/9) in mutants compared with the WT. The *AtQQS‐E* (*P *=* *0.008, 0.009) and *GmNF‐YC4‐1‐OE* (*P *=* *0.00005, 0.00004) mutants showed 70% and 90% less disease, than the WT (Figure 3c).

### Effects of *QQS* expression on Arabidopsis defense are not associated with starch and protein content

**Figure 4:**
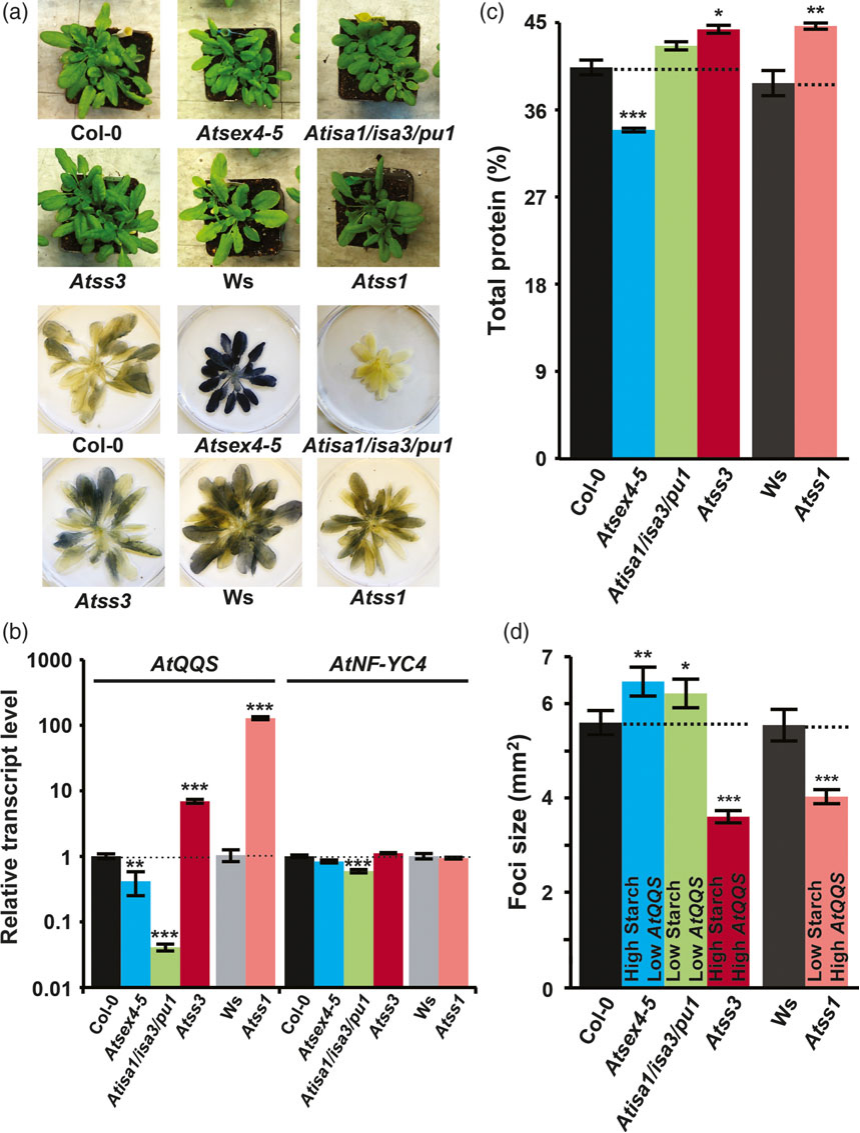
Virus infection of Arabidopsis starch mutants is correlated with *QQS* transcript level. (a) Leaf starch accumulation, (b) the transcript levels of *QQS* and *NF‐YC4* in mutants, quantified by real‐time PCR, and (c) leaf protein content, at the end of light period. (d) The average sizes of TuMV‐GFP infection foci at 120 HAI were increased in plants with down‐regulated *AtQQS* and decreased in plants overexpressing *AtQQS*. Bar charts show mean ± SE,* n *=* *3. ***P <* *0.001; **P <* *0.01; *P <* *0.05.

The importance of carbohydrates, such as sucrose and trehalose, to signalling in response to several biotic stresses (Singh *et al*., 2011; Tauzin and Giardina, 2014) led us to investigate the possible interactions between *QQS* expression level, starch content and pathogen defense. Specifically, because *QQS* overexpression reduces susceptibility to pathogens (Figure 1) and decreases starch content (Li and Wurtele, 2015b; Li *et al*., 2015), we tested whether the decreased susceptibility to TuMV might be coupled to starch content, independent of *QQS* expression level. This investigation was enabled by Arabidopsis starch mutants that represent the four possible combinations of the altered *QQS* transcript level and starch content, as previously determined in plants grown under long‐day (LD) conditions (Table S3): (1) *Atss1* (starch synthase I knockout (Delvalle *et al*., 2005), *QQS* ↑, starch ↓); (2) *Atss3* (starch synthase III knockout (Zhang *et al*., 2005), *QQS* ↑, starch ↑); (3) *Atsex4‐5* (glucan phosphatase knockout (Lu *et al*., 2008), *QQS* ↓, starch ↑) and (4) *Atisa1/isa3/pu1* (a triple knockout of starch debranching enzymes (Wattebled *et al*., 2008), *QQS* ↓, starch ↓). Because our pathogen bioassays were conducted under short‐day (SD) conditions, we tested the composition and level of *QQS* expression for each of these mutants and their corresponding WT controls under SD conditions. Starch accumulation and *QQS* transcript levels in lines under SD conditions (Figure 4a,b) were similar in trend to the same lines under LD conditions (Li *et al*., 2015). When compared with the corresponding WT controls under SD conditions, the protein content was similar in low‐starch mutant *Atisa1/isa3/pu1* (*P *=* *0.6), higher in low‐starch mutant *Atss1* (*P *=* *0.003) and high‐starch mutant *Atss3* (*P *=* *0.029), but lower in high‐starch mutant *Atsex4‐5* (*P *<* *0.001) (Figure 4c). These *QQS* and starch‐perturbed lines were inoculated with TuMV‐GFP. At 5 DPI, the TuMV‐GFP infection foci sizes in the high‐*QQS*‐transcript‐level mutants (*Atss1* and *Atss3*) were 27% and 36% smaller than the WT control (*P *<* *0.001 for both) (Figure 4d). In contrast, the TuMV‐GFP foci in the low‐*QQS*‐transcript‐level mutants (*Atsex4‐5* and *Atisa1/isa3/pu1*) were 16% and 11% larger than the WT control (*P *=* *0.002, <0.05). The starch accumulation, *AtQQS* and *AtNF*‐*YC4* transcript levels were also tested for the *QQS* and *NF‐YC4* mutants under SD conditions (Figure S4a,b). The resistance to TuMV‐GFP increases in plants with higher *QQS* transcript levels and appears to be independent of starch content (Table S3).

### The QQS interaction with Arabidopsis NF‐YC4 and human NF‐YC

**Figure 5:**
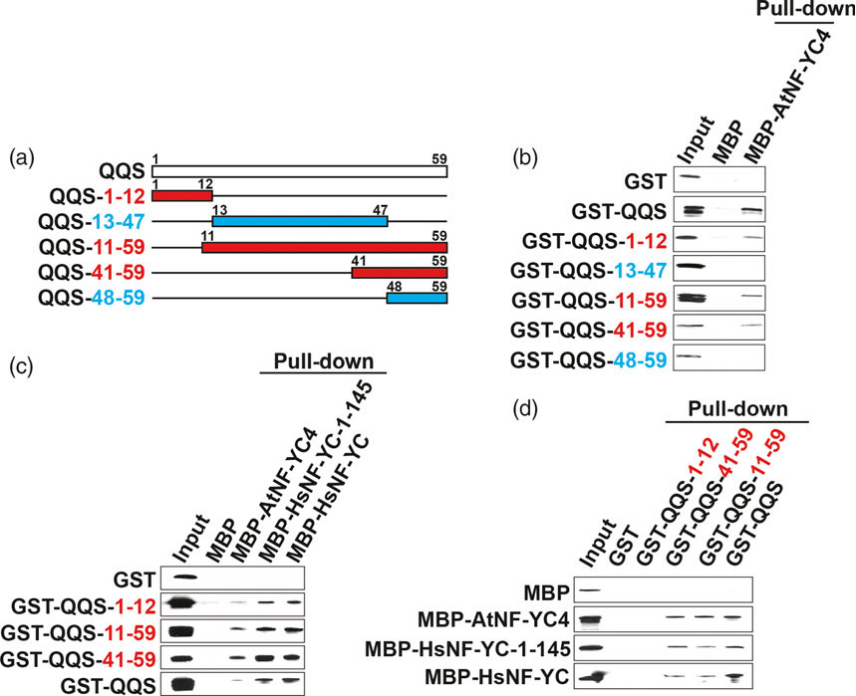
QQS binding with NF‐YC4. (a) QQS fragments used for pull‐down assays. NF‐YC fragments used are shown in Figure S5. (b) MBP pull‐down assays: QQS binds to AtNF‐YC4 in the regions of aa 1‐12 and aa 41‐59. (c) MBP pull‐down assays: QQS binds to HsNF‐YC in the regions of aa 1‐12 and aa 41‐59. (d) GST pull‐down assays: QQS fragments bind to AtNF‐YC4, full‐length HsNF‐YC and the first‐145‐aa N‐terminal of HsNF‐YC. Blue font, no interaction, red font, interaction between QQS fragments and NF‐YC.

Our finding that QQS and NF‐YC4 may play an important role in plant defense led us to investigate the interaction between QQS and NF‐YC in more detail. The heterotrimeric NF‐Y transcription factor complex is conserved across eukaryotic species (Laloum *et al*., 2013; Nardini *et al*., 2013) and modulates gene expression in part via binding to the CCAAT box promoter motif (Nardini *et al*., 2013; Ripodas *et al*., 2014). Our previous study demonstrated that AtNF‐YC4 binds to QQS between aa 73 and 162 (Li *et al*., 2015). Here, we used a computational model to analyse potential QQS/NF‐YC4 interaction sites (Figure S5a). We dissected this potential interaction by screening five fragments of QQS (primers used are in Table S4), selected based on the secondary structure model prediction, for their ability to interact with NF‐YC4 in pull‐down assays (Figure 5a). Pull‐down assays using maltose‐binding protein (MBP)‐NF‐YC fragments as bait indicate that the 12‐N‐terminal aa of QQS (QQS‐1‐12), the 49‐C‐terminal‐aa (QQS‐11‐59) and the 19‐C‐terminal‐aa (QQS‐41‐59) peptides interact with AtNF‐YC4, while the middle‐35‐aa (QQS‐13‐47) and the 12‐C‐terminal‐aa (QQS‐48‐59) peptides do not bind to AtNF‐YC4 (Figure 5b).

To explore the cross‐kingdom universality of the QQS/NF‐YC interactions and to extend the biological significance of the interactions between QQS and NF‐YC, we investigated whether the QQS fragments also interact with human NF‐YC (HsNF‐YC). Protein sequence alignments indicated that the HsNF‐YC shares a conserved H2A domain with AtNF‐YC4; thus, the entire HsNF‐YC as well as the N‐terminal fragment of HsNF‐YC (aa 1‐145, HsNF‐YC‐1‐145) was used (Figure S5b). MBP pull‐down assays indicate there is a physical interaction between all tested QQS fragment peptides (QQS‐1‐12, QQS‐11‐59 and QQS‐41‐59) and the HsNF‐YC‐1‐145 peptide (Figure 5c). Reciprocal Glutathione Sepharose Tag (GST) pull‐down assays using bound QQS fragments as bait show that the QQS‐11‐59 and QQS‐41‐59 peptides pulled down AtNF‐YC4, HsNF‐YC and HsNF‐YC‐1‐145 (Figure 5d). The QQS‐1‐12 fragment failed to pull down any of the NF‐YC proteins (Figure 5d); a possible explanation for this is that the binding site of the 12‐aa QQS‐1‐12 peptide might be masked by the large GST protein moiety on the beads.

Thus, the N‐terminal (QQS‐1‐12) and C‐terminal peptides (QQS‐41‐59) bind to AtNF‐YC4 and HsNF‐YC, but the middle fragment (QQS‐13‐47) does not. The interaction between the C‐terminal‐19‐aa QQS and NF‐YC is stronger than that between the N‐terminal‐12‐aa QQS and NF‐YC.

### Potential QQS interactions with NF‐Y

**Figure 6:**
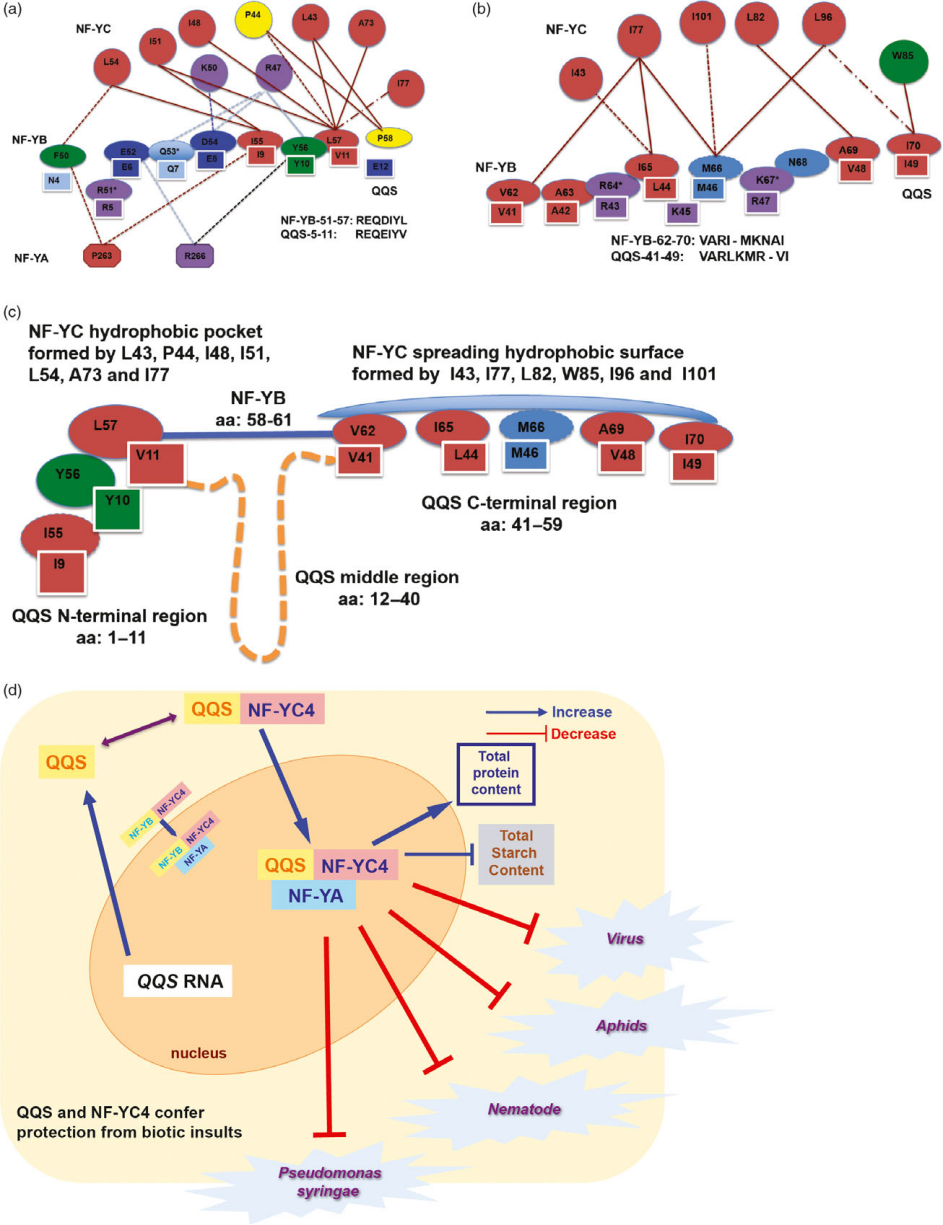
QQS binding sites with NF‐YC and speculated model for QQS and NF‐YC4‐induced changes in composition and plant defense. (a) Model of the interactions of NF‐YB‐51‐57, NF‐YC, and NF‐YA, and the QQS‐5‐11 interactions with NF‐YC. (b) Model of the interactions of NF‐YB‐62‐70 and NF‐YC, and the QQS‐41‐49 interactions with NF‐YC. NF‐YB‐62‐70 are not in contact with NF‐YA, so NF‐YA is not represented here. See Figure S6a,b for detailed explanation of the models, which are based on analysis of previously published crystal structure data [Protein Database Bank (PDB) IDs: 1N1J (Romier *et al*., 2003) and 4 AWL (Nardini *et al*., 2013)]. (c) QQS binds to two NF‐YC hydrophobic interfaces. Based on the sequence similarity between QQS‐5‐11 and QQS‐41‐49 and the NF‐YB N‐terminal region, we propose QQS binds to two NF‐YC hydrophobic interfaces that the NF‐YB N‐terminal region binds to (Figure 6a,b). However, NF‐YB only has three residues (aa from 58 to 61, solid light blue line) in α1 to link the two binding sites and QQS has a 29‐residue long fragment (aa 12–40, dashed yellow line) to link the QQS N‐ and C‐terminal binding sites. Shape of the residues: octagon, NF‐YA; ellipse, NF‐YB; circle, NF‐NC; and shaded rectangle, QQS. Colour of residues represents the polarity and hydrophobicity: red, aliphatic; purple, negatively charged; blue, positively charged; light blue, polar; green, aromatic; and yellow, unique Pro (P). The distance cut‐offs for interaction: 5 Å for hydrophobic interaction, 6 Å for ionic interaction and cation‐pi interaction, and 3.5 Å and 4.0 Å for hydrogen bond when the donor is oxygen/nitrogen and sulphur, respectively. Solid line represents the interaction in both 1N1J and 4 AWL, the broken line in 1N1J only, and the dashed line in 4 AWL only. The colour of the lines represents the type of interaction: dark red, hydrophobic interaction; light blue, hydrogen bond; dark blue, ionic interaction; and black, cation‐pi interaction. The residue marked with * is in contact with DNA within 5 Å. (d) Proposed model of defense priming by QQS: QQS/NF‐YC4 protein complex moves to the nucleus, binding NF‐YA to regulate transcription of downstream genes. QQS may compete with NF‐YB to bind NF‐YC, thus altering the NF‐Y protein complex.

The QQS‐1‐12 and QQS‐41‐59 fragments each bind to AtNF‐YC4 and to human NF‐YC. Furthermore, a sequence alignment of QQS‐1‐12 and QQS‐41‐59 with NF‐YBs identified a 7‐aa consensus motif REQEIYV (QQS‐5‐11) and a 10‐aa motif VARLKMRVI (QQS‐41‐49) that are similar to motifs within NF‐YB in the N‐terminal region near the histone‐binding domain (Figure S5c). The consensus sequences are R[E/D]Q[D/E]‐[Y/F/W][L/V] and [V/I]‐R[L/I]M[K/R]‐[I/V/L]. QQS‐5‐11 aligns to the disordered region and the coil near the N‐terminus (NF‐YB‐51‐57), whereas QQS‐41‐49 aligns to the α1 helix and loop‐1 region (NF‐YB‐62‐70) (Figure 6a,b). Based on computational analyses of the contact maps of NF‐YB‐51‐57 and NF‐YB‐62‐70 in the NF‐YB and NF‐YA dimer, and of the NF‐YA/NF‐YB/NF‐YC trimer and DNA complex (Figure 6a,b), we propose that the QQS N‐terminus and C‐terminus bind to NF‐YC at the same binding sites as NF‐YB‐51‐57 and NF‐YB‐62‐70, and we further propose that the middle region QQS (QQS‐12‐40) may form a loop to bring the two QQS binding sites close to each other (Figure 6c). The structure of the NF‐YA/NF‐YB/NF‐YC and DNA complex in the region of NF‐YB‐51‐57 and NF‐YB‐62‐70 shows that the disordered region located at NF‐YB‐51‐57 and the structured region located at NF‐YB‐62‐70 are buried in the cavity formed by the hydrophobic interface of NF‐YC and hydrophilic interfaces of DNA and NF‐YA (Figure S6). The sequence diversity and structural flexibility in this region of plant NF‐YBs are consistent with this domain providing specific recognition for the NF‐YA/NF‐YB/NF‐YC association and modulating DNA transcription via an interaction with DNA. Taken together, these data lead us to speculate, proposing a model in which QQS binds to NF‐YC, potentially dissociating NF‐YB or preventing NF‐YB binding (Figure 6d). *In vivo* experimentation will be important to validate this model.

Searches of the patented protein sequence database (http://www.ebi.ac.uk/patentdata/proteins) identified nine plant‐related peptides with the motif R[E/D]Q[D/E]‐[Y/F/W][L/V] (QQS‐1‐12 region) and three candidates with the motif [V/I]‐R[L/I]M[K/R]‐[I/V/L](QQS‐41‐49 region), but without histone‐like motifs (Figure S7). (No polypeptides in the database contained both the consensus fragments QQS‐1‐12 and QQS‐41‐49 (Figure S7a,b).) Transgenic plants expressing these sequences were tolerant to an herbicide (Guo *et al*., 2015; Wu *et al*., 2015), but to our knowledge have not been tested for pathogen susceptibility (Table S5a,b). We propose that polypeptides with these motifs may bind to NF‐YC, as QQS does, and modify plant NF‐Y associations and regulate transcription.

## Discussion

*QQS* may be the first orphan gene identified as having a biochemically characterized metabolic function (Li *et al*., 2009). It interacts with a conserved transcription factor, NF‐YC4 (Li *et al*., 2015). Both QQS and NF‐YC4 regulate carbon and nitrogen allocation, affecting starch and protein accumulation in leaves and seeds (Li and Wurtele, 2015b; Li *et al*., 2009, 2015). When *QQS* or *NF‐YC4* is up‐regulated, starch is decreased and protein is increased. The tight linkage of *QQS* expression to environmental stresses and genetic perturbations has led us to the hypothesis that *QQS* provides a homeostatic function and optimizes tolerance to biotic/abiotic perturbations by mediating crosstalk between primary metabolism and environmental changes (Arendsee *et al*., 2014; Li and Wurtele, 2015b; Li *et al*., 2009, 2015).

In Arabidopsis plants, the expression of *QQS*, and to a lesser extent of *NF‐YC4*, is responsive to biotic stimuli and is differently regulated depending on the pathogen (Figure S1, Arendsee *et al*., 2014). For example, *Pst* DC3000 and TuMV are pathogenic to Arabidopsis; *QQS* and *NF‐YC4* transcripts are down‐regulated following exposure to each of these pathogens. In contrast, *P. infestans* is not pathogenic to Arabidopsis; following *P. infestans* inoculation *QQS* and *NF‐YC4* expression is initially repressed, but by 24 HAI it is not. These kinetics fit well with previous observations that *P. infestans* completes its early stages of infection within 6 HAI as it would in a susceptible host, but by 24 HAI, a resistance reaction in the form of a hypersensitive response is activated in Arabidopsis (Huitema *et al*., 2003). Overall, the data indicate that *QQS* expression is down‐regulated by successful pathogens during disease. This is consistent with our observations in transgenic plants that *QQS* overexpression leads to decreased susceptibility. The most likely explanation of why the RNAi and knockout plants are not more susceptible to *Pst* DC3000 than the WT plants is because *Pst* DC3000 has one or more effectors that can suppress the basal defense mechanisms mediated by *QQS* and *NF‐YC4*. Therefore, knocking out the *QQS* or *NF‐YC4* plant genes does not benefit this bacterium. However, the antibacterial effects of defense mediated by QQS and NF‐YC4 are revealed when we use the *Pst* DC3000 ΔCEL mutant, which lacks the ability to transfer key effectors into the plant. *Pst* DC3000 ΔCEL grows more rapidly in mutants with *QQS* or *NF‐YC4* gene expression reduced or eliminated, relative to its growth in WT plants. This indicates that basal defenses may not be as effective in mutant plants underexpressing *QQS* or *NF‐YC4*, allowing a debilitated bacterial pathogen to better colonize the plant.

In *GmNF‐YC4‐1‐OE* soybeans, there is no *QQS*, but soybean immunity is enhanced. Taken together, these data indicate that although expression of *QQS* clearly *promotes* enhancement of plant immunity across several species, the QQS‐NF‐YC4 interaction is not *required* to enhance plant immunity.

The levels of resistance to pathogens and herbivores observed in Arabidopsis and soybean plants overexpressing *QQS* or *NF‐YC4* are consistent with quantitative resistance as opposed to qualitative resistance. Quantitative resistance is typically defined as incomplete resistance that can be broad spectrum, whereas qualitative resistance is generally considered to be complete resistance and is conferred by resistance genes with relatively narrow specificities (Poland *et al*., 2009). The plants overexpressing *QQS* and *NF‐YC4* had significant and reproducible increases in resistance to the pathogens and herbivores tested, and although it was not complete, this resistance may be useful for augmenting qualitative and quantitative resistance traits already present in soybean. Quantitative resistance takes on many forms, and there are several potential mechanisms that include: variants of nucleotide‐binding site leucine‐rich repeat proteins, pattern recognition receptors, loss‐of‐function alleles of susceptibility genes and variation in host metabolism (French *et al*., 2016). Given that plants overexpressing *QQS* and *NF‐YC4* also have increased partitioning of resources into protein versus carbohydrate, we hypothesize that the mechanism underlying quantitative resistance in these plants is related to metabolism.

Disaccharide carbohydrates are important regulators of plant defenses against pathogens (sucrose) and aphids (trehalose) (Singh *et al*., 2011; Tauzin and Giardina, 2014). Furthermore, sugar and starch contents are often modified during plant–microbe interactions (Tauzin and Giardina, 2014). For example, aphid feeding increases trehalose metabolism, sucrose content and starch content of infested Arabidopsis and tomato plants (Singh and Shah, 2012; Singh *et al*., 2011); it has been proposed that trehalose provides a signalling mechanism that enhances the conversion of sucrose to starch and consequently reduces the sucrose available to phloem‐feeding aphids (Singh and Shah, 2012). Thus, a potential explanation of the decreased susceptibility to pathogens/pests in Arabidopsis and soybean plants overexpressing *QQS* and *NF‐YC4* is that defenses are induced as a result of altered plant composition. However, comparisons of Arabidopsis starch mutants with altered *QQS* expression show that decreased susceptibility to TuMV is not associated with the starch content and thus is more likely regulated by *QQS* expression level *independent* of the changes in starch content. The soybean aphid is a specialist feeder that can only colonize soybean and its winter hosts, whereas the green peach aphid is a generalist, adapted to overcome diverse plant defense strategies and colonizes hundreds of different species (Blackman and Eastop, 2000; Tilmon *et al*., 2011); this difference in specificity might explain the difference in efficacy of the QQS pathway against these insects in our assays.

Constitutively activated defenses that are effective against pathogens or herbivores can be costly to plants, resulting in decreased biomass and seed yield (Benedetti *et al*., 2015; Heidel *et al*., 2004; Heil *et al*., 2000; Redman *et al*., 2001). In contrast, induced defenses are much less costly, because they are only deployed when the plant is under attack (Conrath *et al*., 2015). We have previously shown in glasshouse and field studies that overexpression of *QQS* in Arabidopsis, soybean, rice and maize does not affect the growth nor reduce the yield of these plants compared with control plants (Li and Wurtele, 2015b; Li *et al*., 2009, 2015), and overexpression of *NF‐YC4* does not appear to impact the growth. Therefore, the enhanced‐defense phenotype associated with QQS and its interacting factor is not concomitant with a growth cost, as might be expected if *QQS* overexpression activated constitutive defenses. Another line of evidence supporting induced versus constitutive defense in plants with up‐regulated *QQS* is that constitutive defenses to pathogens and herbivores are associated with strong expression of many defense‐related genes (Tian *et al*., 2014; Ward *et al*., 1991). However, in the *QQS‐OE* plants, only a handful of defense‐related genes are minimally induced. This indicates that entire pathogen‐ or herbivore‐induced defense pathways are not constitutively activated by *QQS*. Thus, we propose that the plant immune system is more rapidly induced when *QQS* is overexpressed, leading to decreased susceptibility to pathogens/pests. The broad‐spectrum resistance we observed could indicate that *QQS* or *NF‐YC4* overexpression activates a form of priming, which would render plant defenses more responsive to biotic stresses (Conrath *et al*., 2015).

This research has exciting implications with respect to the concept of the so‐called defense‐versus‐yield trade‐off, which is thought to be due in part to the competition among different metabolic pathways for limited plant resources (Huot *et al*., 2014; Mitra and Baldwin, 2014; Robert‐Seilaniantz *et al*., 2011). Inducible defenses benefit a plant, because when it is not under attack, resources are not allocated to defense and instead support growth and development. Overexpression of *QQS* or *NF‐YC4* has the unusual result that the plant is protected against a range of biotic stresses; however, there is no detected trade‐off between this increased plant defensive ability and growth of the plants. QQS, in part through its interaction with NF‐YC4, appears to function at a nexus that controls the allocation of resources to protein, starch and primes the plant response to biotic stimuli (Arendsee *et al*., 2014; Li *et al*., 2009). We note that in soybean, *GmNF‐YC4* overexpression tended to have a greater effect on improved defense to pathogens/pests than the *AtQQS* expression pointing to a need for additional research to understand the role of *GmNF‐YC4* in soybean defenses.

The increased susceptibility to pathogens in *Atqq*s and *Atnf‐yc4* lines indicates that QQS and NF‐YC4 may be important in plant defense. The small protein QQS, although encoded by an orphan gene unique to the model plant *A. thaliana*, interacts with NF‐YC from as divergent a species as humans. Previous studies indicated that NF‐YB and NF‐YC interact through histone fold motifs (Romier *et al*., 2003). Our data on interaction of QQS and NF‐YC led us to propose two additional interaction motifs (QQS‐5‐11 and QQS‐41‐49), which mediate QQS binding to the N‐terminus of NF‐YC, near NF‐YC histone‐binding domain. These motifs may have been neglected in previous studies because they are unstructured and therefore not manifested in the crystal structures of NF‐Y (Nardini *et al*., 2013). Sometimes two proteins with a completely unrelated sequence can fold into the same structure and complete the same biological function. QQS contains these motifs, which we speculate may alter the ability of NF‐YB to bind to NF‐YC, thus modulating its function.

In conclusion, *QQS* expression is differentially regulated in Arabidopsis after exposure to pathogens, implicating *QQS* as having a role in plant defense. Experiments with transgenic Arabidopsis and soybean lines altered in expression of *QQS* or its interacting partner *NF‐YC4* revealed the ability of each of these genes to confer quantitative protection against pathogens/pests. *QQS* and *NF‐YC4* may control allocation of primary resources to defense, thus linking two highly prized agronomic traits, pathogen resistance and yield. The broad resistance or reduced susceptibility conferred by *QQS* and *NF‐YC4* provides a new model to explore how plant defense mechanisms interconnect with primary metabolism, and how a recently evolved gene can play a role in this process. From a broader vantage point, the study reveals the promise of orphan genes as untapped resources for crop improvement.

## Experimental procedures

### Plant materials

*Arabidopsis thaliana* mutants with perturbed *QQS* or *NF‐YC4* expression have been previously generated and characterized: *AtQQS* RNAi (Li *et al*., 2009), *AtQQS*‐*OE* (Li and Wurtele, 2015b) and *AtNF‐YC4‐OE* (Li *et al*., 2015) transformants in a ecotype Columbia (Col‐0*) background with few trichomes; and T‐DNA knockout mutants *Atqqs* (Li *et al*., 2015) and *Atnf‐yc4* (Kumimoto *et al*., 2010; Li *et al*., 2015) in a Col‐0 background with trichomes. For genotype with more than two independent lines that were verified in our previous studies, one representative line was used in current experiments due to growth chamber space. Arabidopsis mutants in starch metabolism with different levels of *QQS* expression and starch content, including *Atss1* [ecotype Wassilewskija (Ws)], *Atss3*,* Atsex4‐5* and *Atisa1/isa3/pu1* (ecotype Col‐0 for the latter three mutants), are knockout mutants of starch synthase I, III, a plant‐specific glucan phosphatase and triple knockout mutant of starch debranching enzymes (Delvalle *et al*., 2005; Lu *et al*., 2008; Wattebled *et al*., 2008; Zhang *et al*., 2005).

Soybean plants: the GmNF‐YC4‐1 (Glyma06g17780) overexpressing (*GmNF‐YC4‐1‐OE*) and Arabidopsis *QQS*‐expressing (*AtQQS‐E*) soybean lines in the Williams 82 background, expressed under the control of the constitutive cauliflower mosaic virus (CaMV) 35S promoter, were generated previously, and the plant composition and expression level of *QQS* or *GmNF‐YC4‐1* have been quantified (Li and Wurtele, 2015b; O'Conner *et al*., 2018).

Plant selection and growth, RNA‐Seq, TuMV‐GFP inoculation assay, BPMV‐GFP inoculation assay, *Pseudomonas* inoculation assay, Aphid infestation, SCN bioassay, Field SDS experiment, RNA isolation and real‐time PCR, Composition analysis, Mapping the QQS and NF‐YC interaction, Protein expression and purification, Pull‐down assay, and Experiment design and statistical methods are provided in Appendix S1 in Supporting Information.

### Accession numbers

Sequence data from this article can be found under the following accession numbers in The Arabidopsis Genome Information Resource: *QQS* (At3g30720), NF‐YC4 (At5g63470), and in LegumeIP: GmNF‐YC4‐1 (Glyma06g17780).

## Author contributions

L.L. designed the research. M.Q., W.Z., J.D.H., U.K., S.O. and L.L. performed research. X.Z., Y.W., C.D., D. M., D.N., G.C.M., G.L.T., S.A.W. and L.L. analysed data. L.L., E.S.W. and S.A.W. wrote the manuscript with inputs from M.Q., W.Z., X.Z., D.N., G.C.M. and G.L.T.

## Supporting information


**Figure S1** *AtQQS* and *AtNF‐YC4* transcript levels are altered in response to plant pathogens.
**Figure S2** Aphid performance on Arabidopsis *QQS* and *NF‐YC4* mutants.
**Figure S3** SCN female counts were decreased in soybean *AtQQS‐E* and *GmNF‐YC4‐1‐OE* mutants after a single 30‐d nematode generation.
**Figure S4** Starch accumulation, and *AtQQS* and *AtNF‐YC4* transcript levels in Arabidopsis QQS and NF‐YC4 mutants under short‐day conditions.
**Figure S5** QQS and NF‐YC interaction.
**Figure S6** Interaction of the N‐terminal region in structure 4 AWL.
**Figure S7** Searching the QQS‐like protein in patented protein sequence database.
**Table S2** Expression of five genes involved in plant defense have altered expression in plant lines that overexpress or underexpress *QQS*.
**Table S3** Mutants in starch metabolism with altered *QQS* or *NF‐YC4* transcript level, altered starch/protein level and their resistance to pathogens.
**Table S4** Sequences of primers and DNA oligonucleotides used for Figure 5.
**Table S5** Sequence information for selected sequences from Figure S7.
**Appendix S1** Supplementary experimental procedures.

**Table S1** Genes with significant changes in the *QQS‐OE* and *QQS* RNAi mutants. (See separate Excel file.)
