# Supplementary material for: QQS orphan gene and its interactor NF‐YC4 reduce susceptibility to pathogens and pests
Source: Plant Biotechnol J. 2018 Jul 6;17(1):252–63. doi: 10.1111/pbi.12961 (PMC6330549; doi:10.1111/pbi.12961)
Supplement: Supplementary file 1 — Figure S1 AtQQS and AtNF‐YC4 transcript levels are altered in response to plant pathogens. Figure S2 Aphid performance on Arabidopsis QQS and NF‐YC4 mutants. Figure S3 SCN female counts were decreased in soybean AtQQS‐E and GmNF‐YC4‐1‐OE mutants after a single 30‐d nematode generation. Figure S4 Starch accumulation, and AtQQS and AtNF‐YC4 transcript levels in Arabidopsis QQS and NF‐YC4 mutants under short‐day conditions. Figure S5 QQS and NF‐YC interaction. Figure S6 Interaction of the N‐terminal region in structure 4 AWL. Figure S7 Searching the QQS‐like protein in patented protein sequence database. Table S2 Expression of five genes involved in plant defense have altered expression in plant lines that overexpress or underexpress QQS. Table S3 Mutants in starch metabolism with altered QQS or NF‐YC4 transcript level, altered starch/protein level and their resistance to pathogens. Table S4 Sequences of primers and DNA oligonucleotides used for Figure 5. Table S5 Sequence information for selected sequences from Figure S7. Appendix S1 Supplementary experimental procedures. [file PBI-17-252-s001.docx]

**Supporting Information**

for

*QQS* orphan gene and its interactor *NF-YC4* reduce susceptibility to pathogens and pests

Mingsheng Qi^1,a^, Wenguang Zheng^2,a^, Xuefeng Zhao^3^, Jessica D. Hohenstein^4^, Yuba Kandel^1^, Seth O’Conner^2,5^, Yifan Wang^6^, Chuanlong Du^6^, Dan Nettleton^6^, Gustavo C. MacIntosh^4^, Gregory L. Tylka^1^, Eve Syrkin Wurtele^2,7^, Steven A. Whitham^1^, Ling Li^2,5,7,*^

^1^Department of Plant Pathology and Microbiology, Iowa State University, Ames, IA, USA; ^2^Department of Genetics, Development and Cell Biology, Iowa State University, Ames, IA, USA; ^3^Laurence H. Baker Center for Bioinformatics and Biological Statistics, Iowa State University, Ames, IA, USA; ^4^Roy J. Carver Department of Biochemistry, Biophysics and Molecular Biology, Iowa State University, Ames, IA, USA; **^5^**Department of Biological Sciences, Mississippi State University, Starkville, MS, USA; ^6^Department of Statistics, Iowa State University, Ames, IA, USA; ^7^Center for Metabolic Biology, Iowa State University, Ames, IA, USA

^*^Correspondence (Tel +1 662-325-7570; fax +1 662-325-7939; email [liling@biology.msstate.edu](mailto:liling@biology.msstate.edu)).

^a^These authors contributed equally to this work.

The following materials are available in the online version of this article.

**Figure S1** *AtQQS* and *AtNF-YC4* transcript levels are altered in response to plant pathogens.

**Figure S2** Aphid performance on *Arabidopsis* *QQS* and *NF-YC4* mutants.

**Figure S3** SCN female counts were decreased in soybean *AtQQS-E* and *GmNF-YC4-1-OE* mutants after a single 30-d nematode generation.

**Figure S4** Starch accumulation, and *AtQQS* and *AtNF-YC4* transcript levels in *Arabidopsis* QQS and NF-YC4 mutants under short-day conditions.

**Figure S5** QQS and NF-YC interaction.

**Figure S6** Interaction of the N-terminal region in structure 4AWL.

**Figure S7** Searching the QQS-like protein in patented protein sequence database.

**Table S1** Genes with significant changes in the *QQS-OE* and *QQS* RNAi mutants. (See separate Excel file.)

**Table S2** Expression of five genes involved in plant defense have altered expression in plant lines that overexpress or underexpress *QQS*.

**Table S3** Mutants in starch metabolism with altered *QQS* or *NF-YC4* transcript level, altered starch/protein level and their resistance to pathogens.

**Table S4** Sequences of primers and DNA oligonucleotides used for Figure 5.

**Table S5** Sequence information for selected sequences from Figure S7.

**Methods S1** Supplementary experimental procedures. Plant selection and growth, RNA-Seq, TuMV-GFP inoculation assay, BPMV-GFP inoculation assay, *Pseudomonas* inoculation assay, Aphid infestation, SCN bioassay, Field SDS experiment, RNA isolation and real-time PCR, Composition analysis, Mapping the QQS and NF-YC interaction, Protein expression and purification, Pull-down assay, and Experiment design and statistical methods.


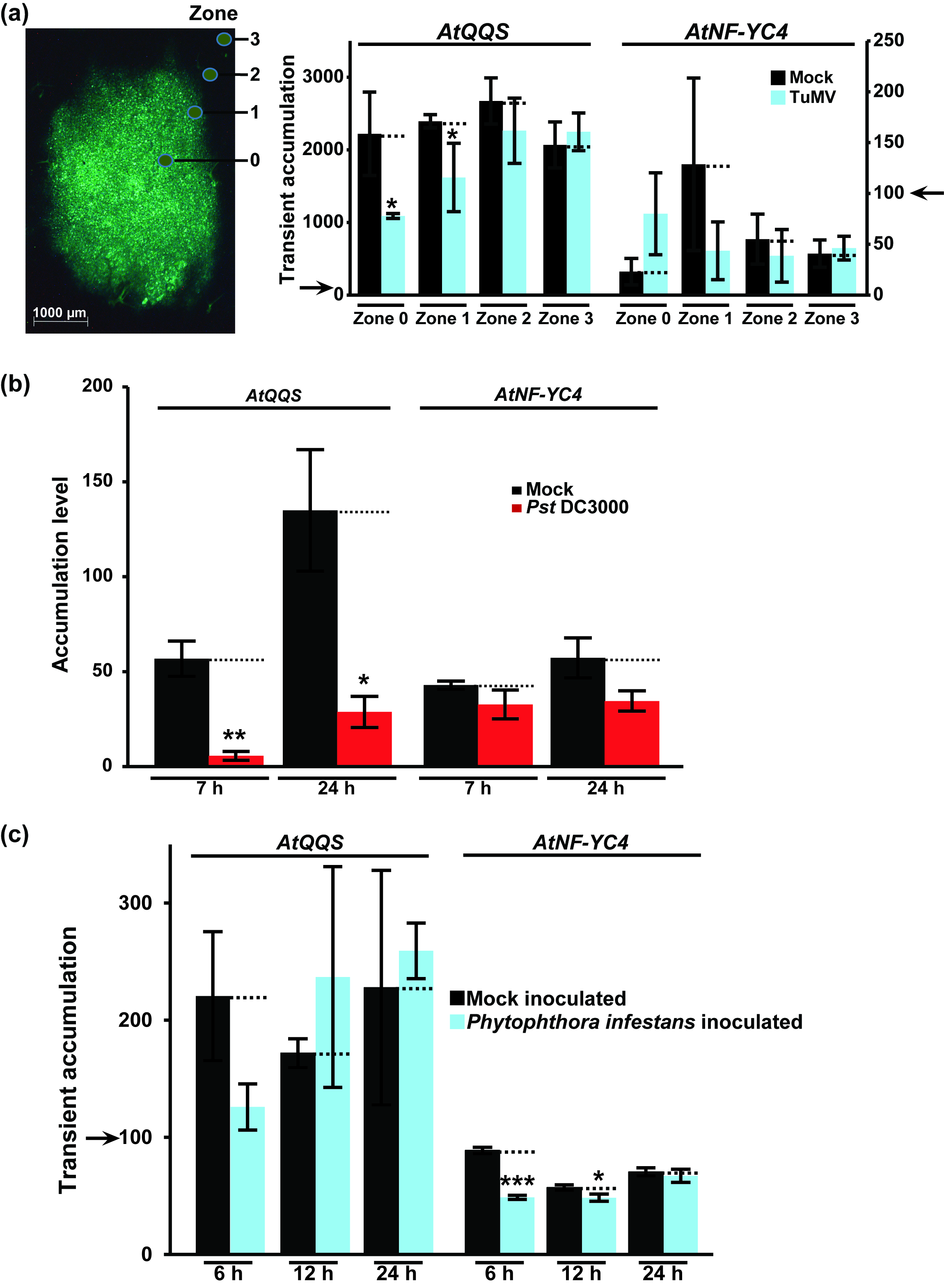


**Figure S1** *AtQQS* and *AtNF-YC4* transcript levels are altered in response to plant pathogens. (a) *QQS* transcript level decreases in TuMV-GFP infected foci. Left**,** sampling locations relative to infection foci. The leaf tissues were sampled at four zones relative to fluorescent TuMV-GFP foci: Zone 0: the epicenter of infection, with strongest GFP fluorescence; Zone 1: adjacent to the center of the infection, with strong GFP signal; Zone 2: periphery of the infection, weak GFP fluorescence; Zone 3: no GFP fluorescence. These zones represent a gradient of TuMV accumulation. Mock infection controls were sampled at same positions relative to mock infection site. Right, *QQS* transcript level decreases in TuMV-infection regions compared to controls (Mock). The microarray data were obtained from ArrayExpress, Experiment ID “E-MEXP-509” (Yang et al., 2007). (b) *QQS* and *NF-YC4* transcript levels decreased in response to *Pseudomonas syringae* DC3000 at 7 h and 24 h after infection. The microarray data was obtained from ArrayExpress, Experiment ID “E-GEOD-5520” (Thilmony et al., 2006). (c) After inoculation with the oomycete *Phytopthora infestans, QQS* and *NF-YC4* transcript levels decrease at 6 h, and *QQS* transcript levels increase at 12 h and 24 h. Five-week-old plants grown at 22°C under 8 h : 16 h (light : dark) were inoculated. The microarray data was obtained from ArrayExpress, Experiment ID “E-GEOD-5616”. Arrow indicates the mean expression level per chip. Bar charts show mean ± SE; *n* = 3. Statistical significance was determined as described in Methods S1 “Experiment design and statistical methods”: ***, *P* < 0.001; **, *P* < 0.01; *, *P* < 0.05.


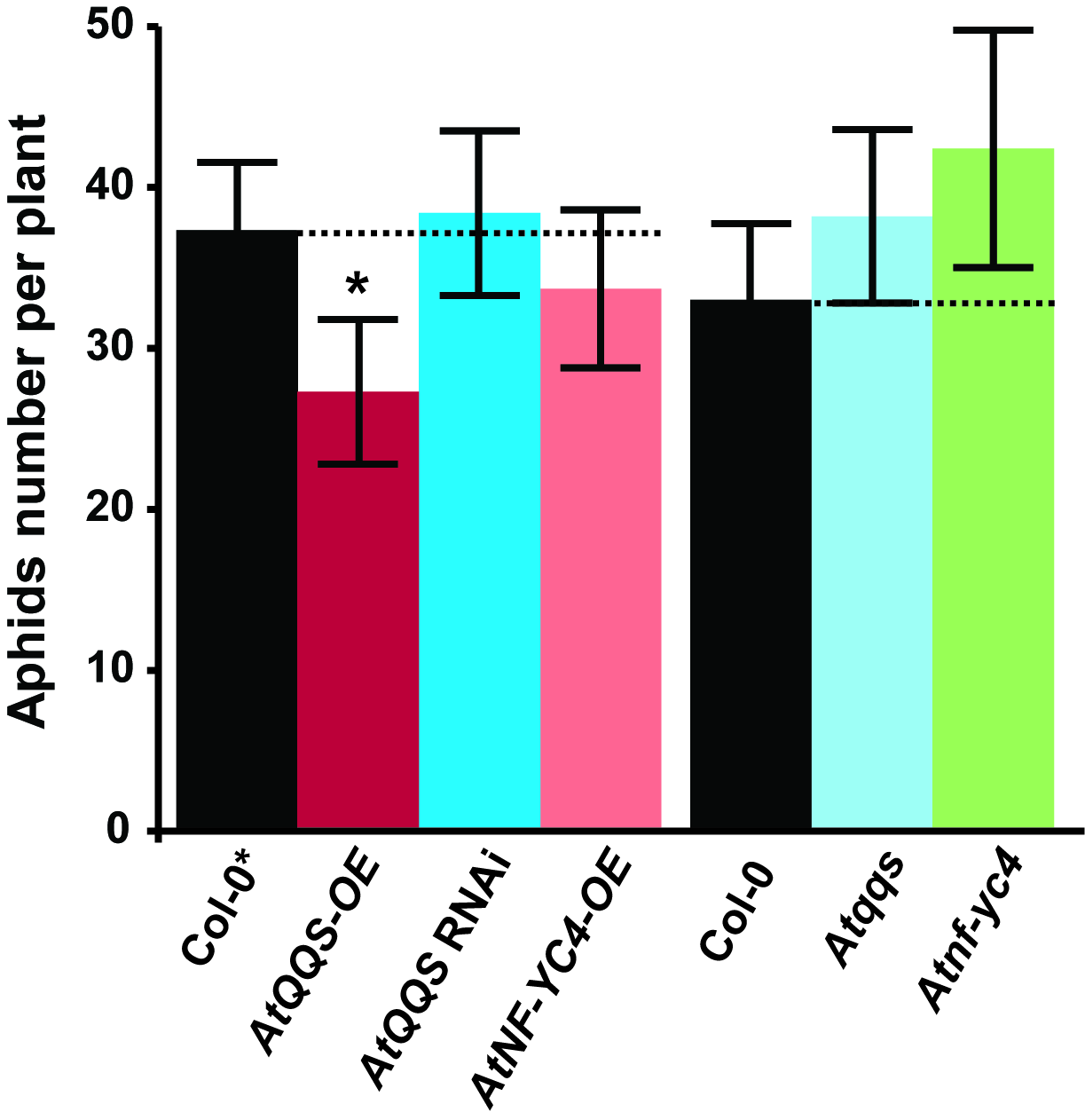


**Figure S2.** Aphid performance on *Arabidopsis* QQS and NF-YC4 mutants. Twenty nine-day-old plants were infested with 10 aphids of adult apterae, 10 plants per line. The number of aphids per plant was counted after seven days. Bar charts show mean ± SE; *n* = 10. *, *P* < 0.05.

**
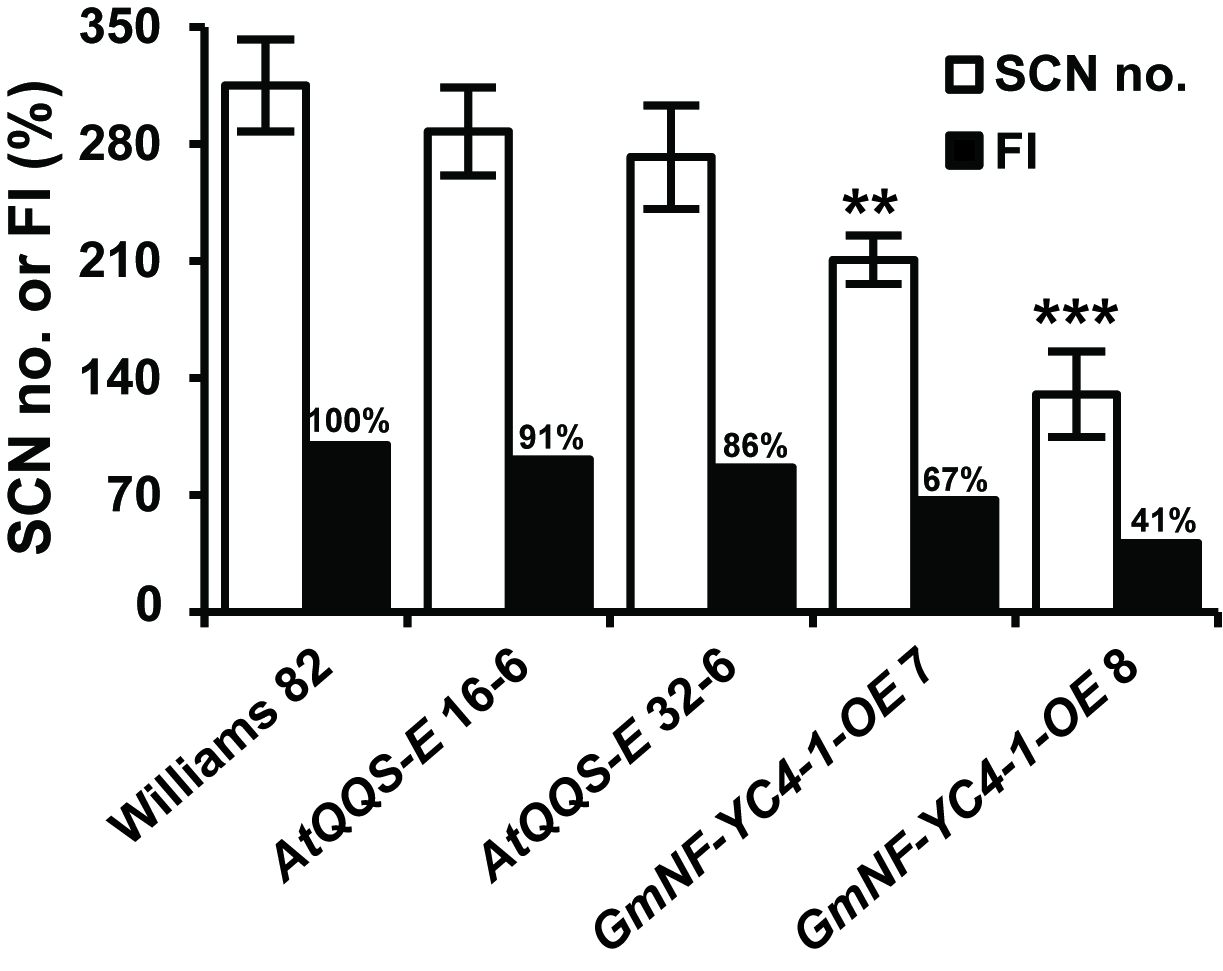
**

**Figure S3** SCN female counts were decreased in soybean *AtQQS-E* and *GmNF-YC4-1-OE* mutants after a single 30-d nematode generation. FI, female index. Williams 82 plants were used as the control. Bar charts show mean ± SE; *n* = 6. ***, *P* < 0.001; **, *P* < 0.01.


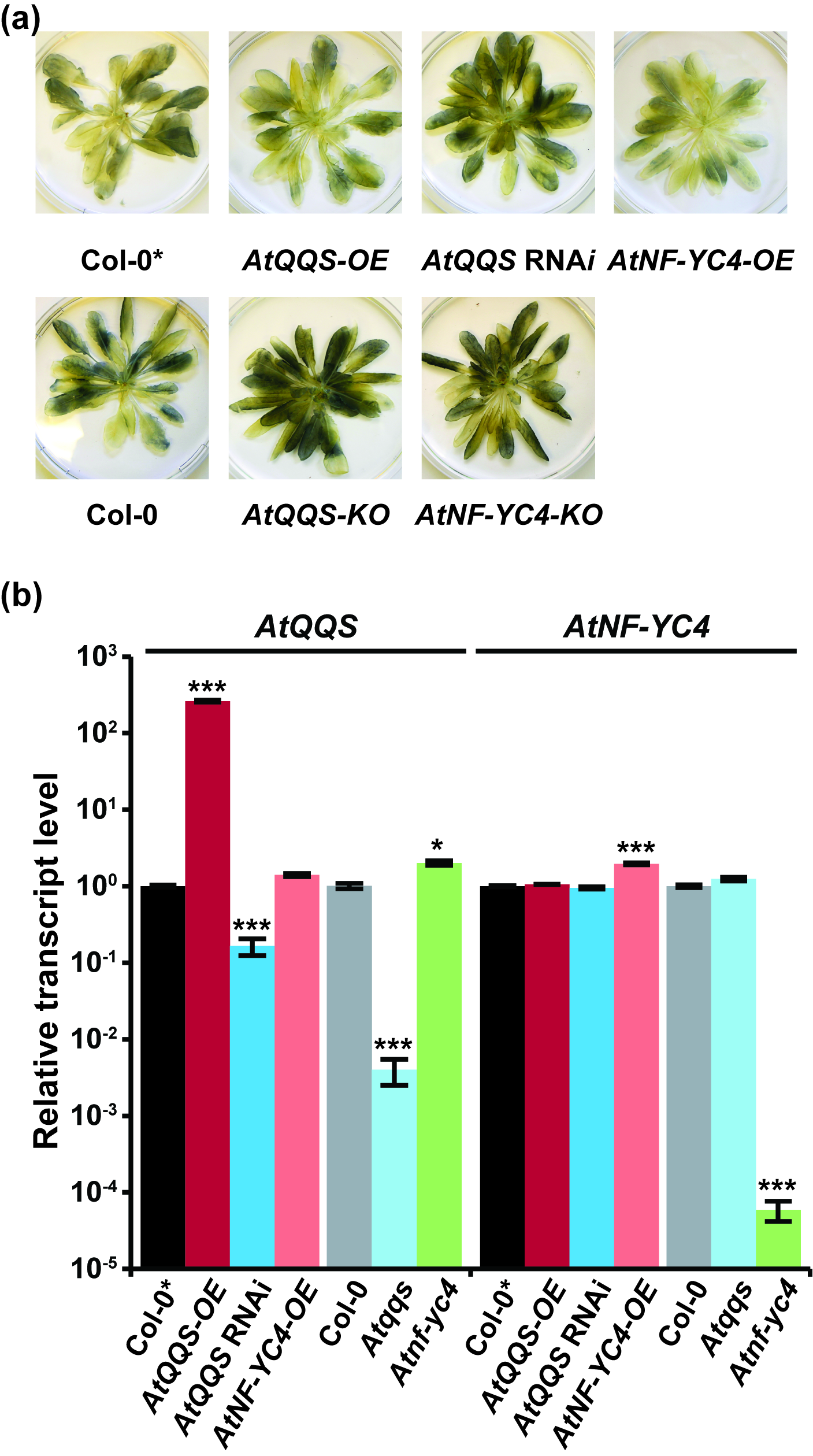


**Figure S4** Starch accumulation, and *AtQQS* and *AtNF-YC4* transcript levels in *Arabidopsis* QQS and NF-YC4 mutants under short-day conditions. (a) Leaf starch accumulation at the end of light period by I_2_/KI staining. (b) The transcript levels of *QQS* and *NF-YC4* in these mutants, quantified by real-time PCR. Bar charts show mean ± SE; *n* = 3. ***, *P* < 0.001; *, *P* < 0.05.


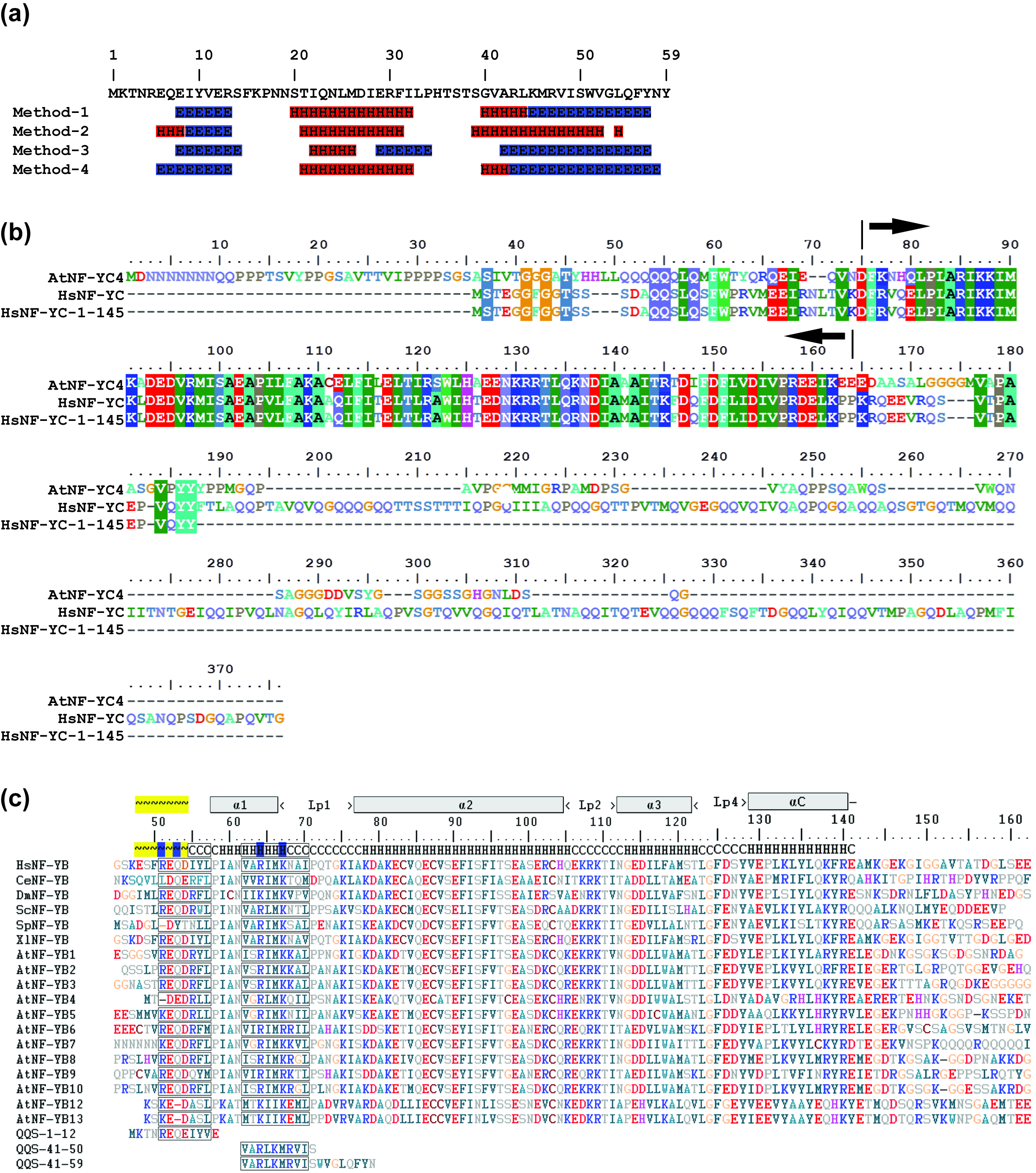


**Figure S5** QQS and NF-YC interaction. (a) Secondary structure prediction of QQS. The secondary structure of QQS was predicted by Quick2D (<https://toolkit.tuebingen.mpg.de/quick2_d>) using various prediction methods. The N-terminal region of aa 5-14, the middle region of aa 20-34 and the C-terminal region of aa 39-58 are predicted to form a β-strand (E), an α-helix (H) and a β-strand (or α-helix), respectively. To search the binding domains of QQS to AtNF-YC4, QQS was dissected into five fragments based on the predicted secondary structures: QQS-1-12 (aa 1-12), QQS-13-47 (aa 13-47), QQS-11-59 (aa 11-59), QQS-41-59 (aa 1-59) and QQS-48-59 (aa 48-59) (Figure 5a). Prediction methods used were: Method-1: divergent profile (PSI-BLAST)-based neural network prediction (PSIPRED, (Jones, 1999)); Method-2: neural network prediction with different types of multiple sequence alignment profiles (Jnet, (Cuff and Barton, 2000)); Method-3 and Method-4: divergent profile-based neural network prediction trained and tested with PSI-BLAST (PROF, (Ouali and King, 2000; Rost, 2001)). (b) Alignment of the protein sequences of *Arabidopsis* NF-YC4, human NF-YC (NP_055038.2) and its N-terminal fragment that were used for pull-down assay in Figure 5. The sequences were aligned with ClustalW and presented using Boxshade (v3.21) software. The dark-shaded boxes indicate amino acid identity, and the lighter-shaded boxes indicate amino acid similarity. Sequence between the arrows is aa 73-162 of NF-YC4 that binds to QQS. (c) Sequence alignments for searching the QQS binding regions. QQS peptides QQS-1-12 and QQS-41-59, which can bind to AtNF-YC4 and Human NF-YC (HsNF-YC), are aligned with NF-YB core regions from human (Hs), *Caenorhabditis elegans* (Ce), *Drosophila melanogaster* (Dm), *Saccharomyces cerevisiae* (Sc), *Schizosaccharomyces pombe* (Sp), *Xenopus laevis* (Xl) and *Arabidopsis thaliana* (At). To search the binding region of QQS-41-59, the non-binding C-terminal peptide QQS-50-59 is trimmed off from QQS-41-59 to form the QQS peptide QQS-41-50, which we propose to physically interact with AtNF-YC4 and HsNF-YC. The AtNF-YC4 interactions were curated by BioGRID (<https://thebiogrid.org>) from the two-hybrid and affinity capture-western experiments (Calvenzani et al., 2012; Hackenberg et al., 2012), all subunits of the AtNF-YB family except AtNF-YB13 were reported to physically interact with AtNF-YC4; however, the binding affinity varies from low to high. The histone-like motifs of HsNF-YB, CeNF-YB, DmNF-YB, ScNF-YB, SpNF-YB, and XlNF-YB were compared by Romier *et al.* (Romier et al., 2003), these sequences are included for comparison of the two boxed regions outside of the histone-like motif: the N-terminal disordered region (aa 48-54), highlighted in yellow in the secondary structure, and the region between the α-helix 1 and the loop 1 (aa 62-70). The secondary structure derived from the crystal structure (PDB ID: 1N1J) is displayed above the sequence alignment (C for coil, H for helix, and ~ for the proposed disordered region due to no density map (Romier et al., 2003)). The four α-helices (α1, α2, α3 and αC) for histone-like motif are shown in the shaded boxes, and the three loop regions (Lp1, Lp2 and Lp3) are marked. The residues in contact with DNA in the crystal structure of the NF-YA/NF-YB/NF-YC and DNA complex (PDB ID: 4AWL (Nardini et al., 2013)) are highlighted in light blue in the secondary structures. The boxed regions are proposed to be the QQS binding sites of QQS-1-12 and QQS-41-59, respectively. The consensus sequences for those two boxed regions are R[E/D]Q[D/E]-[Y/F/W][L/V] and [V/I]-R[L/I]M[K/R]-[I/V/L].

NF-Y function has been intensely studied in humans, where it modulates gene expression (Nardini et al., 2013; Ripodas et al., 2014) and may induce epigenetic modifications (Moeinvaziri and Shahhoseini, 2015). Peptide drugs provide selective influences on cellular processes that can mediate disease in humans; the 2011 peptide market was $14 billion worldwide, with over 60 peptides used as pharmaceuticals, and over 140 more peptides in clinical trials (Fosgerau and Hoffmann, 2015). Because NF-Y is involved in recruiting various proteins to modify cell cycle (Benatti et al., 2011) and disease-related transcriptional activation and repression including cancers (Dai et al., 2015; Kato et al., 2009), hematopoietic disorders (Zhu et al., 2012), and fetal β-hemoglobinopathies (de Souza Carrocini et al., 2015), QQS-type peptides may have the potential to provide general or selective influences on these NF-Y-mediated processes. We propose that QQS may provide a peptide for drug design to target NF-YC. These data support the concept of screening orphan genes as a novel source for pharmaceutical peptides. The interaction of QQS with human NF-YC protein indicates a potential of this small orphan protein to have applications in the treatment of human diseases as well as in crop improvement.


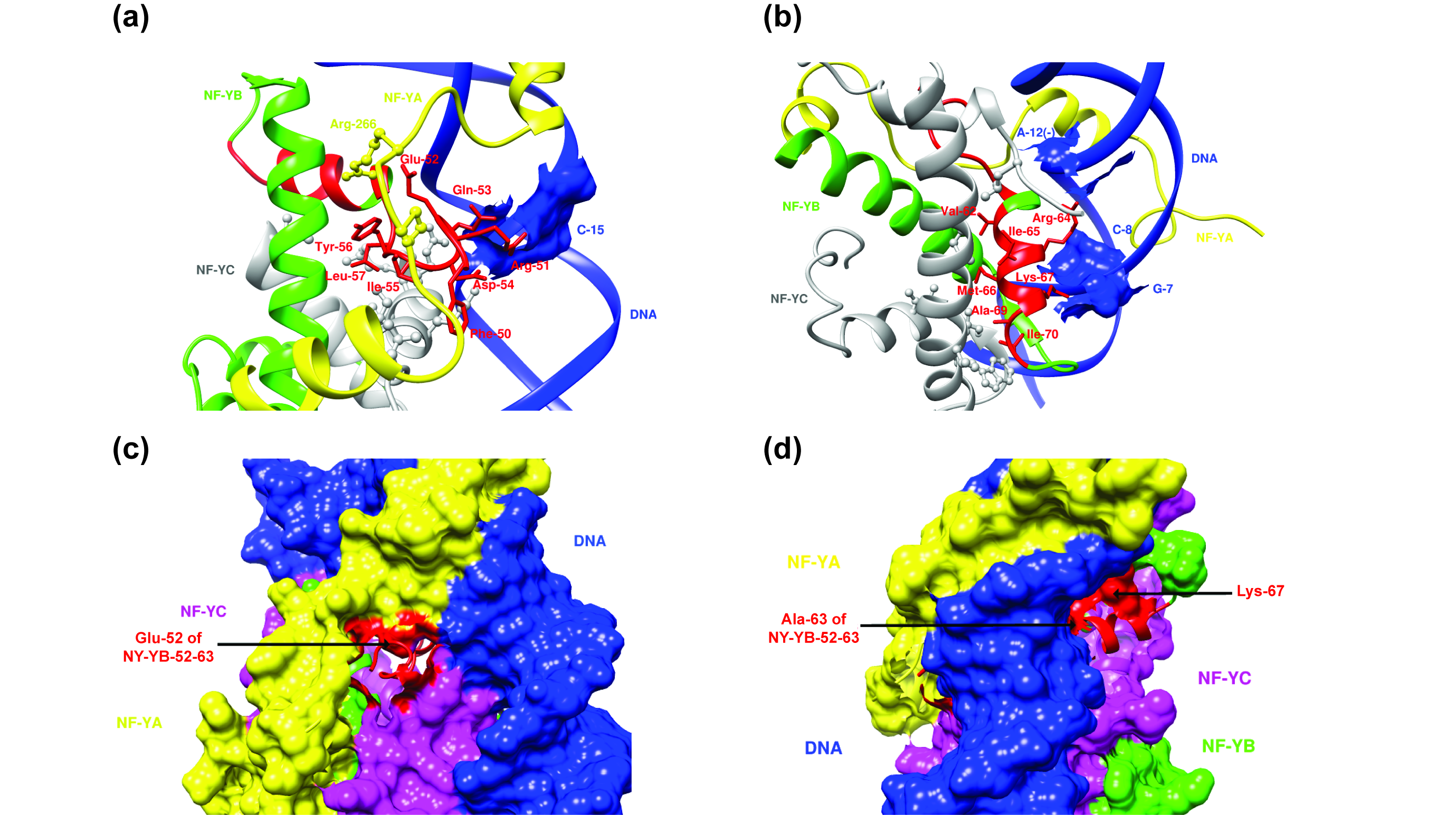


**Figure S6** Interaction of the N-terminal region in structure 4AWL. Interactions of NF-YB-50-57 and NF-YB-62-70 in 4AWL are illustrated in Figure S6a,b. The overall association is displayed in Figure S6c,d (DNA: blue, NF-YA: Yellow, NF-YB: Green, NF-YB-51-57: Red, NF-YB-62-70: Red, NF-YC: grey, side chains of NF-YA/NF-YC: ball and stick for NF-YA, side chains of NF-YB: stick). (a) Interactions of NF-YB-51-57 with NF-YA, NF-YC and DNA. Tyr-56, Leu-57 and Ile-55 of NF-YB fall in the hydrophobic pocket formed by Leu-43, Pro-44, Leu-45, Ile-48, Ile-51, Leu-54, and Ala-73. Arg-51 and Gln-53 are in contact with DNA Cytonine-15 (C-15). Tyr-56 forms hydrogen bond with Arg-47 of NF-YC and has a cation-pi interaction with Arg-266 of NF-YA. (b) Interactions of NF-YB-62-70 with NF-YC and DNA. No contact between NF-YB-62-70 and NF-YA is identified; however, the hydrophobic side chains of Val-62, Ile-65, Met-66, Ala-69 and Ile-70 from two α-helix turns of the eight residues face the hydrophobic interfaces formed by Val-62, Ile-65, Met-66, Ala-69 and Ile-70 of NF-YC. Lys-67 contacts with Guanine-7 (G-7) and Cytosine-8 (C-8), and Arg-64 with Adenine-12 (-) (A-12(-)). Notes for Figure 6a,b that show specific recognition among NF-YA/NF-YB/NF-YC outside of the histone-like motif. The interaction of two NF-YB N-terminal regions NF-YB-51-57 (aa 51-57, Figure 6a) and NF-YB-62-70 (aa 62-70, Figure 6b) with NF-YC and NF-YA is calculated using Protein Interactions Calculator (<http://pic.mbu.iisc.ernet.in> (Tina et al., 2007)) in the crystal structures of the NF-YB/NF-YC dimer (PDB ID: 1N1J) and the NF-YA/NF-YB/NF-YC and DNA complex (PDB ID: 4AWL) (octagon for NF-YA, ellipse for NF-YB, and circle for NF-NC). NF-YB-51-57, which aligns perfectly with the consensus region of QQS-1-12 (aa 5-11, Figure 6a, shaded rectangle for QQS), contains the tetra-peptide (aa 51-54) in the disordered N-terminal region and tri-peptide (aa 55-57) in the coiled region; and NF-YB-62-70, which aligns with the consensus region of QQS-41-50 (aa 41-49, Figure 6b) with one gap, contains six residues from α1 (aa 62-67) and three from Loop1 (aa 68-70). Notes for Figure 6a, the hydrophobic contacts of the tetra-peptide IYLP of NF-YB (aa 55-58, NF-YB-55-58) with NF-YC in 1N1J is almost as the same as those contacts in 4AWL, and the tri-peptide EQD of NF-YB (aa 52-54, NF-YB-52-54) only interacts with NF-YC through hydrogen bonds and ionic bridge upon NF-YA binding to the NF-YB/NF-YC dimer. It is indicated that the highly conserved large hydrophobic residue L-57 of NF-YB is in the hydrophobic pocket formed by five residues of NF-YC in 1N1J and 4AWL. NF-YA P263 is in hydrophobic contact with NF-YB-51-57 and NF-YA R266 forms hydrogen bond with NF-YB E52 and cation-pi interaction with NF-YB Y56. Comparing the observed contacts in 1N1J and 4AWL, we propose that the hydrophobic contact between the tetra peptide IYLP of NF-YB-55-58 plays a major role in NF-YB and NF-YC recognition in this region, and charged tri-peptide EQD of NF-YB-52-54 is important for NF-YA to associate with the NF-YB/NF-YC dimer. An aromatic residue is highly conserved at the position 56 of NF-YB, which implies that the cation-pi interaction between NF-YA R266 and the aromatic residue at the position 56 of NF-YB is another key factor in the NF-YA/NF-YB/NF-YC association. Notes for Figure 6b: NF-YB-62-70 is not in contact with NF-YA, and the hydrophobic side chains of five residues of NF-YB V-62, I-65, M-66, A-69 and I-70 point to the hydrophobic pocket formed by I-43, I-77, L-82, W-85, L-96 and I-101 of NF-YC. The side chains of the highly conserved R-64 and K-67 of NF-YB are in contact with DNA. The consensus sequence [V/I/M]-[R/K][I/L][M/I][K/R]-[I/L/M] in the region of the α1 and Loop1 (Figure S5c) forms a hydrophobic interface on one side of the α1 helix, and negative charged interface on another side, this unique interfaces on α1 is responsible for the association of NF-YB, NF-YC and DNA in this region. QQS-41-49 aligns with NF-YB-62-70 with one gap, we propose that QQS-41-59 binds to AtNF-YC4 and HsNF-YC in the same hydrophobic pocket of NF-YC that NF-YB-62-70 binds to. (c, d) N-terminal region of NF-YB is buried in the cave formed by NA-YA, NF-YC, and DNA. Upon DNA associating with the NF-YA/NF-YB/NF-YC complex, NF-YB-52-63 is in the cave formed by the hydrophobic interface by NF-YB, and by the hydrophobic interfaces by NF-YA and DNA. To illustrate the cave, the surface views of the NF-Ys and DNA complex are shown in Figure S6c,d with the two end residues Glu-52 and Ala-63 labeled, and other residues of NF-YB-52-63 are in the cave. NF-YB-64-70 (aa 64-70) is outside of the cave, but forms a sandwich with NF-YC and DNA. The labeled Lys-67 is in contact with DNA. The buried fragment NF-YB-52-63 contains three residues in the disordered N-terminus (aa 52-54), four residues in the coil at the beginning of the N-term (aa 55-58), and five residues at the beginning of α-1 (aa 59-63). Combining NF-YB-52-63 in the buried cave and NF-YB-64-70 in the sandwich region to form the fragment NF-YB-50-70 (aa 50-70), which contains the two NF-YC binding sites (NF-YB-51-57 and NF-YB-62-70), we propose: 1. the sequence diversity and the structure flexibility of NF-YB-50-70 make it another fingerprint region of NF-YB for the specific NF-YB/NF-YC/NF-YA recognition besides histone-binding motif of NF-Ys; 2. NF-YB-50-70 modulates the NF-YA/NF-YB/NF-YC complex to bind to DNA, and affects its transcription function; 3. the fingerprint region of NF-YB-50-70 is another factor that regulates the selective association among various isoforms of NF-Ys in plant.


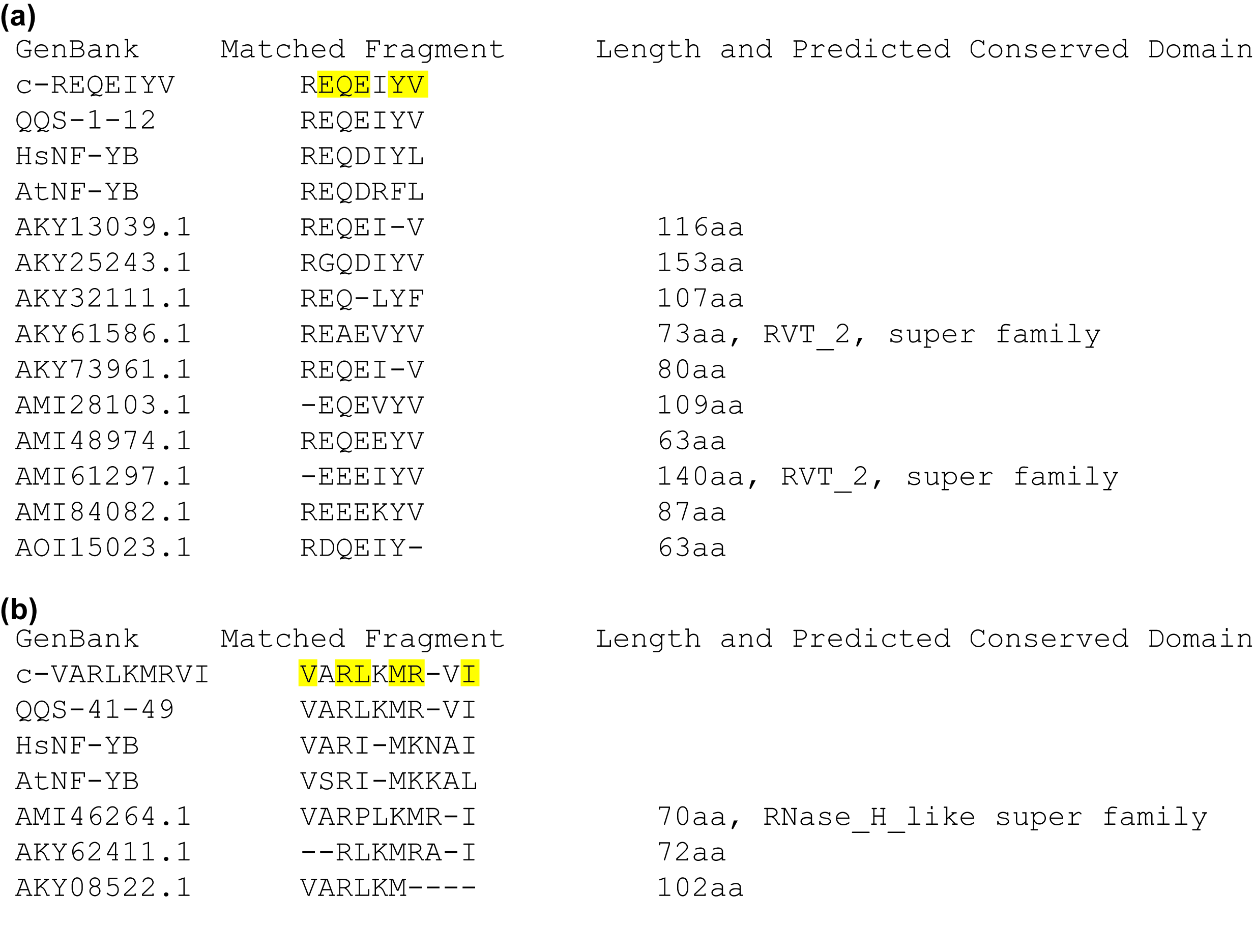


**Figure S7** Searching the QQS-like protein in patented protein sequence database. (a) Sequence alignment of the matched fragments with c-REQEIYV. (b) Sequence alignments of the selected fragments with cVARLKMRVI. After protein blasting the sequences of QQS-5-12 and QQS-41-49 using patented protein sequence database (<https://blast.ncbi.nlm.nih.gov>), the matched sequences were further selected using three criteria: 1. the sequence is related to a plant; 2. the sequence length is less than 150 aa; 3. the sequence is predicted not to be a histone-like protein in conserved domain and protein classification (<https://www.ncbi.nlm.nih.gov/Structure/cdd/cdd.shtml>). The matched fragments from the selected sequences are aligned with the sequence REQEIYV (c-REQEIYV, Figure S7a), the isoform of the consensus sequence R[E/D]Q[E/D]-[Y/F/W][L/V], and VARLKMRVI (cVARLKMRVI, Figure S7b), and the isoform of the consensus sequence [V/I]-R[L/I]M[K/R]-[I/V/L].

**Table S1**. Genes with significant changes in the *QQS-OE* and *QQS* RNAi mutants.

(a) Transcripts that exhibited significant variation in *QQS-OE* plants compared to the WT control plants. (b) Transcripts that exhibited significant variation in *QQS* RNAi plants compared to the WT control plants. (c) Pathways that are over-represented among the transcripts that exhibited significant variation in *QQS-OE* plants, by MetNet Online "Over-representation Search". (See separate Excel file.)

**Table S2** Expression of five genes involved in plant defense have altered expression in plant lines that overexpress or underexpress *QQS*.

|  |  | **Mean of read counts (two to three replicates)** | | | **Ratio** | | ***P* value** | |
| --- | --- | --- | --- | --- | --- | --- | --- | --- |
| **Locus** | **Gene** | ***QQS* RNAi** | ***QQS-OE*** | **WT (Col-0*)** | ***QQS* RNAi vs WT** | ***QQS-OE* vs WT** | ***QQS* RNAi vs WT** | ***QQS-OE* vs WT** |
| **AT1G19640** | **JMT jasmonic acid carboxyl methyltransferase** | **34.5** | **35** | **19.7** | **1.75** | **1.78** | **0.06** | **^*^0.03** |
| **AT1G32640** | **MYC2 transcription factor MYC2** | **323.5** | **439** | **285.7** | **1.13** | **1.54** | **0.67** | **^*^0.04** |
| AT1G43160 | RAP2.6 ethylene-responsive transcription factor RAP2-6 | 4.5 | 11.5 | 1.3 | 3.38 | 8.63 | 0.45 | 0.09 |
| **AT1G56650** | **PAP1 transcription factor MYB75** | **117** | **119.5** | **82.3** | **1.42** | **1.45** | **^*^0.04** | **^*^0.01** |
| AT1G64280 | NPR1 Regulatory protein NPR1 | 113 | 89 | 112.3 | 1.01 | 0.79 | 0.87 | 0.18 |
| AT1G80840 | WRKY40 putative WRKY transcription factor 40 | 20 | 36.5 | 18.3 | 1.09 | 1.99 | 0.89 | 0.07 |
| AT2G14610 | PR1 pathogenesis-related protein 1 | 128.5 | 620.5 | 307.3 | 0.42 | 2.02 | 0.16 | 0.19 |
| AT2G26020 | PDF1.2b putative defensin-like protein | 1 | 9.5 | 4.3 | 0.23 | 2.19 | 0.14 | 0.19 |
| AT2G43790 | MPK6 MAP kinase 6 | 169.5 | 125.5 | 162 | 1.05 | 0.77 | 0.87 | 0.09 |
| **AT3G05500** | **AT3G05500 Rubber elongation factor protein; stress related protein** | **74.5** | **102.5** | **70.7** | **1.05** | **1.45** | **0.9** | **^*^0.04** |
| AT3G14620 | CYP72A8 cytochrome P450, family 72, subfamily A, polypeptide 8 | 304.5 | 311 | 316 | 0.96 | 0.98 | 0.67 | 0.81 |
| **AT4G01720** | **WRKY47 putative WRKY transcription factor 47** | **19.5** | **15.5** | **35** | **0.56** | **0.44** | **^*^0.01** | **^*^0.00** |
| AT5G22570 | WRKY38 putative WRKY transcription factor 38 | 7.5 | 29.5 | 18 | 0.42 | 1.64 | 0.06 | 0.14 |

Significant change is considered as ^*^, *P* < 0.05. Data from Li *et al.* 2015 (Li et al., 2015). Blue font indicates significantly differential accumulation.

**Table S3** Mutants in starch metabolism with altered *QQS* or *NF-YC4* transcript level, altered starch/protein level and their resistance to pathogens.

| **Genotype** | **Gene.Name** | **LocusID** | **Background** | **SD starch** | **SD protein** | **SD QQS transcript level change (%)** | **SD NF-YC4 transcript level change (%)** | **SD virus foci size change (%)** | **SD virus foci # change (%)** | **SD virulent bacterial # change (%)** | **SD non-virulent bacterial # change (%)** | **Under LD conditions** | **Starch change (%) LD** | **QQS transcript level change (%) LD** |
| --- | --- | --- | --- | --- | --- | --- | --- | --- | --- | --- | --- | --- | --- | --- |
| ***ss3*** | *SS3* | At1g11720 | Col-0 (trichomes) | Increased | Increased | 588 | 11 | -36 | -- | -- | -- | Starch increase, QQS up | 22 | 15 |
| ***sex4-5*** | *SEX4* | At3g52180 | Col-0 (trichomes) | Increased | Decreased | -58 | -16 | 16 | -- | -- | -- | Starch excess,QQS down | 126 | -77 |
| ***isa1/isa3/pu1*** | *ISA1/ISA3/PU1* | At2g39930/At4g09020/At5g04360 | Col-0 (trichomes) | Decreased | Increased | -96 | -40 | 11 | -- | -- | -- | Starch decrease,QQS down | -99 | -100 |
| **Col-0** | -- | -- | Col-0 (trichomes) | -- | -- | -- | -- | -- | -- | -- | -- | -- | -- | -- |
| ***qqs*** | *QQS* | At3g30720 | Col-0 (trichomes) | Increased | Decreased | -100 | 24 | 52 | 46 | 31 | 207 | Starch increase | 20 | -- |
| ***nf-yc4*** | *NF-YC4* | At5g63470 | Col-0 (trichomes) | Similar | Similar | 100 | -100 | 28 | 17 | 18 | 102 | Similar | Similar | -- |
| ***ss1*** | *SS1* | At5g24300 | Ws | Decraesed | Increased | 12174 | -7 | -27 | -- | -- | -- | Starch decrease, QQS up | -23 | 170 |
| **Ws** | -- | -- | Ws | -- | -- | -- | -- | -- | -- | -- | -- | -- | -- | -- |
| ***QQS* RNAi** | *QQS* | At3g30720 | Col-0 (few trichomes) | Increased | Decreased | -84 | -5 | 52 | 12 | 42 | 174 | Starch  increase | 30 | -- |
| ***QQS-OE*** | *QQS* | At3g30720 | Col-0 (few trichomes) | Decraesed | Increased | 26074 | 5 | -32 | -22 | -63 | -14 | Starch decrease, QQS up | -15 | 14270 |
| ***NF-YC4-OE*** | *NF-YC4* | At5g63470 | Col-0 (few trichomes) | Decraesed | Increased | 40 | 96 | -51 | -88 | -88 | -7 | Starch decrease | -14 |  |
| **Col-0*** | -- | -- | Col-0* (few trichomes) | -- | -- | -- | -- | -- | -- | -- | -- | -- | -- | -- |

**Table S4** Sequences of primers and DNA oligonucleotides used for Figure 5.

| **Primer/DNA oligonucleotide** | **Sequence** |
| --- | --- |
| QQS-1-12-F | 5'-GATCCATGAAGACCA ATAGAGAGCA GGAAATTTACGTTGAAG-3' |
| QQS-1-12-R | 5'-AATTCTTCAACGTAAATTTCCTGCTCTCTATTGGTCTTCATG-3' |
| QQS-48-59-F | 5'-GATCCGTCATATCATGGGTCGGGCTTCAGTTCTACAACTACTGAG-3' |
| QQS-48-59-R | 5'-AATTCTCAGTAGTTGTAGAACTGAAGCCCGACCCATGATATGACG-3' |
| QQS-41-59-F | 5'-GATCCGTCGCAAGGCTCAAAATGAGGGTCATATCATGGGTCGGGCTTC AGTTCTACAACTACTGAG-3' |
| QQS-41-59-R | 5'-AATTCTCAGTAGTTGTAGAACTGAAGCCCGACCCATGATATGACCCTCATT TTGAGCCTTGCGACG-3' |
| QQS-11-59-F | 5'-CCCGGATCCGTTGAAAGAAGCTTCAAACCAAAC-3' |
| QQS-11-59-R | 5'-CCCGAATTCTCAGTAGTTGTAGAACTGAAGCCC-3' |
| QQS-13-47-F | 5'-CCCGGATCCAGAAGCTTCAAACCAAACAATTCA-3' |
| QQS-13-47-R | 5'-CCCGAATTCTCACCTCATTTTGAGCCTTGCGAC-3' |
| HsNF-YC-F | 5'-CCCGGATCC ATGTCCACAGAAGGAGGATTTGGT-3' |
| HsNF-YC-R | 5'-CCCCTCGAG TCAGTCGCCGGTCACCTGGGGGGC-3' |

**Table S5** Sequence information for selected sequences from Figure S7.

No sequence is identified to align with both QQS-1-12 and QQS-41-49, all selected sequences in Figure S7a,b were patented for tolerance from exposure to an herbicide, and the seeds of the transgenic plants which comprises the recombinant DNA are stable.

**Table S5a** Sequences that align with QQS-1-12.

| **GenBank** | **Sequence Information from NCBI** (https://www.ncbi.nlm.nih.gov/protein) |
| --- | --- |
| AKY13039.1  AKY25243.1  AKY32111.1  AKY61586.1  AKY73961.1 | Sequences from **Patent US 9012723**. The sequence numbers in the patent are 100346 (AKY13039.1), 112550(AKY25243.1), 119418(AKY32111.1), 148527 (AKY61586.1), and 160902 (AKY73961.1) respectively.  Authors: Guo,L., Kovalic,D.K., Qiu,B.-X., Tabaska,J.E. and Wu,W.  Title: Isolated novel acid and protein molecules from soy and methods of using those molecules to generate transgene plants with enhanced agronomic traits.  Patent: US 9012723-B2 160902 21-APR-2015;  Monsanto Technology LLC; St. Louis, MO  CAMBIA Patent Lens: US 9012723  Major claims in **Patent US** 9012723 (<http://patft.uspto.gov>):  1. The transgenic plant with the recombinant DNA is homozygous, and expresses a protein that provides tolerance from exposure to an herbicide such as glyphosate, dicamba, or glufosinate compound. 2. The seeds of the transgenic plants corn, soybean, cotton, canola, alfalfa, wheat, rice, sugarcane, or sugar beet, which comprises the recombinant DNA, are stable. |
| AMI28103.1  AMI48974.1  AMI61297.1  AMI84082.1 | Sequences from **Patent** **US 9029636**. The sequence numbers in the patent are 149337 (AMI28103.1), 170208 (AMI48974.1), 182531 (AMI61297.1), and 205316 (AMI84082.1) respectively.  Authors: Wu,W., Tabaska,J., Kovalic,D., Qiu,B.-X. and Guo,L.  Title: Isolated novel nucleic acid and protein molecules from soy and methods of using those molecules to generate transgenic plants with enhanced agronomic traits    Patent: US 9029636-B2 205316 12-MAY-2015;  Monsanto Technology LLC; St. Louis, MO  CAMBIA Patent Lens: US 9029636  Major claims in **Patent** [9029636](http://patft.uspto.gov/netacgi/nph-Parser?patentnumber=9029636) (<http://patft.uspto.gov>): 1. The transgenic plant with the recombinant DNA provides tolerance from exposure to an herbicide (glyphosate, dicamba, or glufosinate compound) applied at levels that are lethal to a wild type of plant. 2. The seeds of the transgenic plants corn, soybean, cotton, canola, alfalfa, wheat, rice, sugarcane, or sugar beet, which comprises the recombinant DNA, are stable. |

**Table S5b** Sequences that align with QQS-41-49.

| **GenBank** | **Sequence Information** |
| --- | --- |
| AKY62411.1  AKY08522.1 | Sequences from **Patent** **US 9012723**. The sequence numbers in the patent are 149352(AKY62411.1) and 95829 (AKY08522.1).  Authors: Guo,L., Kovalic,D.K., Qiu,B.-X., Tabaska,J.E. and Wu,W.    Title: Isolated novel acid and protein molecules from soy and methods of using those molecules to generate transgene plants with enhanced agronomic traits    Patent: US 9012723-B2 149352 21-APR-2015;  Monsanto Technology LLC; St. Louis, MO  CAMBIA Patent Lens: US 9012723  Major claims in **Patent US 9012723**: See Table S5a. |
| AMI46264.1 | Sequence 167498 from patent US 9029636.  Authors: Wu,W., Tabaska,J., Kovalic,D., Qiu,B.-X. and Guo,L.  Title: Isolated novel nucleic acid and protein molecules from soy and methods of using those molecules to generate transgenic plants with enhanced agronomic traits.  Patent: US 902936-B2 167498 12-MAY-2015;  Monsanto Technology LLC; St. Louis, MO  CAMBIA Patent Lens: US 902936  Major claims in **Patent US 9029636**: see Table S5a. |

# Methods S1 Supplementary experimental procedures

**Experimental procedures**

Plant selection and growth

*Arabidopsis* plants were grown in a growth chamber at 22 ^o^C under LD or SD conditions as described (Li et al., 2009a). The seeds were planted on petri dishes, those harboring a “BAR” gene were selected with glufosinate as previously described (Jones et al., 2016), and plants were transferred to Sunshine Mix LC1 soil in pots at 12 d after planting. Soybean transformants expressing *AtQQS* (*AtQQS-E*) or overexpressing *GmNF-YC4-1* (*GmNF-YC4-1-OE*) were identified by PCR analysis for presence of the *QQS* or *GmNF-YC4-1* via vector specific primers as described before (Li and Wurtele, 2015). The vector specific primers are pB2GW7-F: 5'-ACATTACAATTTACTATTCTAGTCGA-3' and pB2GW7-R: GCGGACTCTAGCATGGCCG-3'; the control-gene primers for soybean are 18S-rRNA-F: 5'-GGGCATTCGTATTTCATAGTCAGAG-3' and 18S-rRNA-R: 5'-CGGTTCTTGATTAATGAAAACATCCT-3'.

RNA-Seq

Total RNA was extracted from pooled *Arabidopsis* leaf samples of *QQS-OE*, *QQS* RNAi and Col-0* at the end of light period under LD conditions in a growth chamber, purified, and sent to BGI Americas for sequencing as previously described (Li et al., 2015). The RNA-Seq data were deposited in the NCBI Sequence Read Archive (<https://www.ncbi.nlm.nih.gov/sra/>), accession number: SRP072425.

TuMV-GFP inoculation assay

TuMV-GFP was inoculated on *Arabidopsis* plants as described before (Yang et al., 2007) with minor modifications. Frozen TuMV-GFP-infected turnip leaves (cultivar Seven Top) were ground in 20 mM sodium phosphate buffer (pH 7.2, 1:6, wt : vol) and filtered through Miracloth (Calbiochem, San Diego, CA, USA) to obtain the inoculum. The titer of the inoculum was adjusted to yield well-separated GFP foci.

The *Arabidopsis* plants were grown for 7 weeks under 10 h : 14 h (light : dark) at 22 ºC to allow large rosette leaves to develop. Rosette leaves were dusted with Carborundum and rubbed to inoculate with TuMV-GFP using a cotton-stick applicator (Yang et al., 2007). At 120 HAI, GFP foci on the inoculated rosette leaves were counted under UV illumination (100-W Blak-Ray longwave UV lamp; UVP, Upland, CA, USA). Each genotype had three biological replicates of ten randomly selected plants. The average foci number of 10 plants of each genotype was determined and the significance of foci number differences between lines was determined as described in Statistical Methods.

For each genotype, 40 single GFP foci were randomly selected and photographed with a Zeiss Stemi SV11 fluorescence dissecting microscope using a Zeiss AxioCam MRc5 digital camera (Hewezi et al., 2008). The resulting digital files were processed using Zeiss Axiovision software. Each photographed GFP focal area was processed with the ImageJ measure tool (<http://imagej.nih.gov/ij/>) and calibrated against the correct scaling of the original image from the Stemi SV11. The total area for the GFP focus was calculated as square millimeters. The individual measurements for the GFP foci of each genotype were used to calculate an average focus size for each genotype tested. Statistical significance of size differences between lines was determined as described in Statistical Methods.

BPMV-GFP inoculation assay

Frozen BPMV-GFP-infected soybean leaves (Williams 82) were ground in 50 mM sodium phosphate buffer (pH 7.2) and filtered through Miracloth (Calbiochem, San Diego, CA, USA) to obtain the inoculum.

Soybean *AtQQS-E*, *GmNF-YC4-1-OE* and Williams 82 control plants were grown in a growth chamber under 14 h : 10 h (light : dark), at 22 ^o^C, for 14 days. The plant genotypes were confirmed by PCR screening of leaf DNA as described in (Li and Wurtele, 2015). The primary leaves were dusted with Carborundum and rubbed to inoculate with BPMV-GFP using gloved-finger. At 11 and 13 DAI, GFP foci on the third trifoliate leaves were observed and recorded under UV illumination (100-W Blak-Ray longwave UV lamp; UVP, Upland, CA, USA). The statistical significance of the difference in virus infection levels between each mutant plant line and its corresponding WT was determined as described in Statistical Methods, *n* = 3 biological replicates (three lines from three independent transformation events of *AtQQS-E* and *GmNF-YC4-1-OE*) with 6 plants per replicate.

*Pseudomonas* inoculation assay

Assays are a modification of the method of Katagiri *et al.* (Katagiri et al., 2002). *Arabidopsis* plants were grown for 32 d in a growth chamber under 10 h : 14 h (light : dark), at 22 ^o^C. *P. syringae* bacterial cultures were centrifuged, washed with buffer, re-centrifuged, and re-suspended in inoculation buffer (10 mM MgCl_2_, 0.05% Silwet L-77). Plants were sprayed with a bacterial inoculum with the bacteria level adjusted to 10^8^ CFU mL^-1^. Bacterial levels *in planta* were determined by cutting leaf disks with a cork borer (inner diameter 0.5 cm) and completely homogenizing them in 500 μl of the inoculation buffer. The resulting suspension containing the bacteria was diluted and plated on King’s B Medium (proteose peptone, 20 g l^-1^; K_2_HPO_4_, 1.5 g l^-1^; MgSO_4_, 1.5 g l^-1^; glycerol, 10 ml l^-^; agar, 15 g l^-1^) plates with rifampicin (50 µg ml^-1^). Numbers of bacteria were evaluated at 0 DPI (Day 0) and 4 DPI (Day 4). The statistical significance of the difference in bacterial level between each mutant plant line and its corresponding WT was determined as described in Statistical Methods, *n* = 3 biological replicates with 4 plants per replicate.

Soybean *AtQQS-E*, *GmNF-YC4-1-OE* and Williams 82 control plants were grown in a growth chamber under 14 h : 10 h (light : dark), at 22 ^o^C. The plant genotypes were confirmed by PCR screening of leaf DNA as described in (Li and Wurtele, 2015). The first trifoliate leaves on 23-d-old soybeans were inoculated. Freshly cultured *Psg*R4 was re-suspended in inoculation buffer to the final concentration of around 10^7^ CFU ml^-1^. The leaflets of each trifoliate leaf were pricked by needle before 5 μl of the inoculum was placed onto each wound (ten per leaflet). Bacterial levels *in planta* were determined at 7 DPI in a way similar to the *Arabidopsis* plants, *n* = 3 biological replicates (three lines from three independent transformation events of *AtQQS-E* and *GmNF-YC4-1-OE*) with 4 plants per replicate.

Aphid infestation

*Plant Material and Growth Conditions:* *Arabidopsis* seeds of Col-0*, *QQS* RNAi, *QQS-OE*, and *NF-YC4-OE*; Col-0, *nf-yc4*, and *qqs*, were planted directly in soil in pots that were 4 inches in diameter and 3.5 inches in height, at 21 ^o^C, under 16 h : 8 h (light : dark). Soybean seeds from two lines from two independent transformation events of *AtQQS-E* and *GmNF-YC4-1-OE* and Williams 82 (the control) were grown in pots at 25 ^o^C, under16 h of light/8 h of dark. Herbicide was sprayed to *QQS* RNAi, *QQS-OE*, and *NF-YC4-OE* *Arabidopsis* plants, and *AtQQS-E,* and *GmNF-YC4-1-OE* soybean plants to identify the mutants.

*Experimental Design and Statistical Analysis:* Experiments were originally set up in a randomized complete block design with 10 pots per flat (each with one plant). Plants in one flat were infested before moving onto the next flat, ensuring similarly-sized aphids on all 10 plants. However, if some plants did not germinate or had significant aphid death as evidenced during counting, these plants were not included in the statistical analyses. Thus, the final statistical design was a randomized incomplete block design.

*Arabidopsis thaliana:* Green peach aphids (*Myzus persicae* Sulzer) were obtained from a laboratory colony maintained on bok choy plants at Iowa State University. Basal rosette leaves of 29-d-old *Arabidopsis* plants were infested with ten mixed-age apterous aphids using a fine-tip paintbrush. These aphids were confined using clip cages (BioQuip products 1458, Rancho Dominguez, CA, USA); they were allowed to feed and reproduce on the plants for 7 d, and populations were then counted, as previously described (Hillwig et al., 2016), with 10 biological replicates.

*Glycine max*: Soybean aphids (*Aphis glycines* Matsumura; biotype 1) were obtained from a laboratory colony at Iowa State University; aphids were maintained on the aphid-susceptible genotype SD01-76R. Either one unifoliate leaf or one leaflet on the first trifoliate of 18-d soybean plants was infested with ten mixed-age apterous aphids using a fine-tip paintbrush. These aphids were confined using clip cages and populations were counted after 7 d, in two lines from two independent transformation events of *AtQQS-E* and *GmNF-YC4-1-OE*, with 8 biological replicates.

SCN bioassay

The reproduction of SCN on the soybeans was assessed by following the methods of Niblack *et al.* (Niblack et al., 2009) for assessing soybean resistance to SCN in the greenhouse. Briefly, soybean seeds of each genotype (two lines from two independent transformation events of *AtQQS-E* and *GmNF-YC4-1-OE*, Williams 82 (control, susceptible variety), and Jack (the positive control with resistance)) were planted directly into individual replicate cone-tainers filled with soil-sand mix infested with SCN HG Type 2.5.7 (Niblack et al., 2002) originally obtained from Muscatine, Iowa. All cone-tainers were placed in randomly determined positions in buckets of sand in a greenhouse water bath and incubated at constant 27 °C under natural and supplemented lighting conditions. The plant genotypes were confirmed by PCR screening of leaf DNA as described in (Li and Wurtele, 2015).

After 30 d of incubation, enough time for a single SCN generation, the soil and roots from each individual cone-tainer were carefully removed, then the soil from each root system was carefully washed away. The roots were subsequently placed on a sieve with 850 µm pores nested over a sieve with 250 µm pores and sprayed with a strong stream of water. The stream of water dislodged the SCN females from the roots. The SCN females passed through the top, 850-µm-pore sieve and were collected on the bottom, 250-µm-pore sieve. The SCN females collected on the bottom sieve and all other debris on that sieve were observed with a dissecting microscope, and the number of SCN females recovered from each individual plant was counted. *n* = 6 biological replicates.

A female index was calculated for each plant genotype by dividing the number of SCN females formed on the plant by the number of SCN females formed on Williams 82, a susceptible soybean variety, then multiplying by 100. The statistical significance of the difference between each mutant plant line and its corresponding WT was determined as described in Statistical Methods.

Field SDS experiment

Soybean plants, two lines from two independent transformation events of *AtQQS-E* and *GmNF-YC4-1-OE* and Williams 82 controls, were planted at Iowa State University Research Farm on 26 May 2017. Each treatment was planted in six replicates in a randomized complete block design. Herbicide was sprayed to soybean leaves to identify the *AtQQS-E*, and *GmNF-YC4-1-OE* mutants. The experiment was conducted in a field with the history of SDS, plus SDS inoculum was included with soybean seed at a rate of 8.3g/m of linear row at planting. The *F. virguliforme* isolate, *NE305*, was originated locally and isolated from a single spore and grown on sterile white sorghum seed following the previously published protocols (de Farias Neto et al., 2006; Li et al., 2009b). Sprinkler irrigation was set up and the field was watered 2-3 times a week depending upon rainfall to create a more favorable environment for disease development.

Field plots were visited weekly starting at growth stage R3 (beginning of pod formation) and foliar SDS symptoms began to appear, and assessments were made, at R5 growth stage (Fehr et al., 1971). Incidence of the disease (DI) was estimated on percentage (0-100%) based on number of plants with foliar symptoms per row out of total plants. Disease severity (DS) was assessed on a 1-9 scale using a previously published SDS rating scale (Gibson et al., 1994; Kandel et al., 2015) that was based on chlorotic or necrotic area on leaf and premature defoliation, where 1 = 1-10% leaf surface chlorotic or 1-5% necrotic and 9 = premature plant death. Using DI and DS, foliar disease index (FDX) was calculated as: FDX = disease incidence (DI) × disease severity scale (DS)/9. *n* = 6 biological replicates.

RNA isolation and real-time PCR

The 39-d-old *Arabidopsis* seedlings at the end of light period under SD conditions were used for extraction of RNA; RNAs were treated with DNase I and purified as previously described (Li et al., 2015). Two μg of RNA and SuperScript^®^ III First Strand kit (Invitrogen, Carlsbad, CA, USA) were used for cDNA synthesis. Quantitative real-time PCR (qRT-PCR) was performed using the cDNA and gene-specific primers of *QQS* (QQS-F: 5'-ATGAAGACCAATAGAGAGCAGGA-3' and QQS-R: 5'-TTTTGAGCCTTGCGACACCTGATGT-3') and *NF-YC4* (NF-YC4-F: 5'-ATG GACAATAACAACAACAACAACAACC-3' and NF-YC4-R: 5'-CGGTGACTATTGATGCAGATCC-3'). Each cDNA was amplified by quantitative PCR using iQ^™^ SYBR^®^ Green Supermix (Bio-Rad, Hercules, CA, USA) and iCycler real-time PCR system (Bio-Rad). *At18S rRNA* (primers: At18R-F: 5'-GGGCATTCGTATTTCATAGTCAGAG-3' and At18R-R: 5'-CGGTTCTTGATTAATGAAAACATCCT-3') were used for *Arabidopsis*, as the reference gene to normalize the expression value in each sample.

Composition analysis

Seedlings of *Arabidopsis* were harvested for leaf starch and protein tests at the end of the light period from *Arabidopsis* plants grown in soil in a growth chamber at 39 d after planting under SD conditions. I_2_/KI staining for starch and determination of protein content were conducted as previously described (Li et al., 2015). *n* = 3 biological replicates.

Mapping the QQS and NF-YC interaction

Aligning QQS sequence against all protein sequence and structure databases (Non-redundant protein sequences, Reference proteins, UniprotKB/Swiss-Prot, Patented protein sequences, Protein Data Banks proteins, Metagenomic proteins, Transcriptome Shotgun Assembly proteins) (<http://blast.ncbi.nlm.nih.gov/>), no homologous protein was identified, and QQS sequence shows extremely low similarity to other protein sequences. The protein secondary structure prediction server Quick2D (<http://toolkit.tuebingen.mpg.de/quick2_d>) summarizes the prediction results from multiple prediction servers. Although the prediction results from different secondary structure prediction servers do not agree with each other completely, they have consensus and indicate that the fragment EIYVER(aa 8-13) at the N-terminus is predicted to fold into a beta sheet, the fragment TIQNLMDIERFI (aa 21-32) in the middle into a helix, and the fragment GVA (aa 40-42) into a short helix, and the fragment RLKMRVISWVGLQFY (aa 43-57) at the end of the C-terminus into a long beta sheet. To map the interaction between QQS and AtNF-YC4 and HsNF-YC, based on the predicted secondary structure, QQS was fragmented into five peptides QQS-1-12, QQS-13-47, QQS-11-59, QQS-41-59 and QQS-48-59.

Protein expression and purification

The coding sequences for aa 11-59 (QQS-11-59) and aa 13-47 (QQS-13-47) of QQS were amplified by PCR using diluted pGEX-2T-QQS plasmid DNA as template and the primer pairs listed in Table S4. The coding sequences for aa 1-12 (QQS-1-12), aa 48-59 (QQS-48-59), aa 41-59 (QQS-41-59) of QQS were synthesized by annealing complementary pairs of oligonucleotides (Table S4), respectively. The PCR products and the synthesized DNA fragments were cloned into pGEX-2T-1 vector (GE Healthcare) using BamH I and EcoR I sites. The coding sequences for HsNF-YC (NP_055038.2) and its truncated N-terminal, aa 1-145 (HsNF-YC-1-145) were amplified by PCR and cloned into MBP-H vector using BamH I and Xho I sites. The DNA template for HsNF-YC was purchased from OriGene (Rockville, MD, USA; Cat# RC20060). All the constructs were verified by sequencing and transformed into BL21 (DE3) cells. For the protein expression, the cells were grown at 37 °C until OD600 reaches 0.5-0.7 and induced with 0.1 mM IPTG at 16°C for 16 h. Cells were harvested by centrifugation at 6000 X *g* for 15 min.

The cells expressing the GST and GST-tagged recombinant proteins were re-suspended in lysis buffer (50 mM Tris pH7.5, 150 mM NaCl, and 0.05% NP-40), and lysed by incubation with 0.25 mg/mL lysozyme for 0.5-1 h followed by sonication using Qsonica Sonicator Q125 (40% amp, 10 sec on, 10 sec off, for 2 min). The supernatants were incubated with Glutathione Sepharose 4 Fast Flow beads (GE Healthcare, Little Chalfont, UK) at 4°C for 2 h with gentle rotation. The beads were washed with lysis buffer and elution buffer (50 mM Tris pH8.0) sequentially at 4°C. The beads were stored at 4 °C in the elution buffer or eluted with 10 mM reduced glutathione in the elution buffer at 4 °C. The MBP and MBP-tagged recombinant proteins were purified using amylose resin (New England Biolabs, Ipswich, MA, USA) following the manufacturer’s instructions.

Pull-down assay

The purified proteins were quantified with BSA (bovine serum albumin) standards based on the signal intensity on SDS-PAGE gels. About 3-5 μg of each of the immobilized bait protein and the input protein was incubated in 800 μl of GST lysis buffer above at 4 °C for 4 h with gentle rotation. The beads were washed with lysis buffer 4 times, and a final wash with GST elution buffer. The input proteins and pull-down samples were boiled with SDS loading buffer and loaded on 15% SDS-PAGE gel for separation. The blots were immunoblotted with anti-MBP antibody (New England Biolabs) or anti-GST antibody (Sigma, St. Louis, MO, USA) for GST pull-down assay or MBP pull-down assay, respectively.

Experiment design and statistical methods

A randomized complete block design was used when plants were grown, collected and analyzed. A minimum of three biological replicates from each genotype and control were used.

Data displayed in Figure 1b, 4b, and 4d were analyzed using a linear model with fixed effects for blocks, fixed effects for genotypes, and random errors assumed to be normally distributed with constant variance. As part of each linear model analysis, an F-test was conducted for each comparison of interest. The *P* values from these F-tests were used to determine statistical significance.

Data displayed in Figure 3c, 4c and S4 were analyzed as in Figure 1b, 4b, and 4d, except that values of the response variable were log transformed prior to analysis to make the constant error variance assumption viable. Data corresponding to Figure 2b were logged and values from technical replicates averaged before proceeding with the linear model analysis used for Figure 1b, 4b, and 4d.

Data displayed in Figure 1a, 3a, 3b, S2, and S3 were analyzed by fitting a generalized linear model with a Poisson response and log mean modeled as a linear function of fixed block effects and fixed genotype effects. As part of each generalized linear model analysis, Wald tests were used to determine significance for Figure 1a, and F-tests adjusted for overdispersion were used to determine significance for Figure 3a, 3b, S2, and S3.

Data displayed in Figure 2a were analyzed, separately for each time point, by fitting a generalized linear model with a binomial response. The logit of the success probability was modeled as a linear function of replication and genotype effects. The number of binomial trials was set at 6 to match the number of plants evaluated for each combination of replication and genotype. Wald tests were used to determine *P* values for each genotype comparison.

For the data in Figure 1c, log counts were averaged over technical replicates, and a linear model was fit to these average log counts. The linear model included fixed effects for replications and all combinations of treatment, genotype, and days after infection. As part the linear model analysis, an F-test was conducted for each comparison of interest. The *P* values from these F-tests were used to determine statistical significance.

Log normalized expression levels of *QQS* and *NF-YC4* were used as response variables for the analyses of the data displayed in Figure S1. For Figure S1a,b, a linear model was fit to each response, with fixed effects for replications, fixed effects for HAI, fixed effects for treatments (infection or mock), and fixed effects for treatment x HAI interactions. As part of each linear model analysis, F-tests for treatment effects at each hour after infection were conducted, and *P* values from those F-test were used to determine statistical significance. The analysis for Figure S1c was similar except that zone was used in place of HAI and the linear model included a random effect for each combination of replication and treatment to account for the whole-plot experimental units in the split-plot design used in the experiment associated with Figure S1c.

**References**

Benatti, P., Dolfini, D., Vigano, A., Ravo, M., Weisz, A. and Imbriano, C. (2011) Specific inhibition of NF-Y subunits triggers different cell proliferation defects. *Nucleic acids research* **39**, 5356-5368.

Calvenzani, V., Testoni, B., Gusmaroli, G., Lorenzo, M., Gnesutta, N., Petroni, K., Mantovani, R. and Tonelli, C. (2012) Interactions and CCAAT-binding of Arabidopsis thaliana NF-Y subunits. *PLoS One* **7**, e42902.

Cuff, J.A. and Barton, G.J. (2000) Application of multiple sequence alignment profiles to improve protein secondary structure prediction. *Proteins* **40**, 502-511.

Dai, C., Miao, C.X., Xu, X.M., Liu, L.J., Gu, Y.F., Zhou, D., Chen, L.S., Lin, G. and Lu, G.X. (2015) Transcriptional activation of human CDCA8 gene regulated by transcription factor NF-Y in embryonic stem cells and cancer cells. *J Biol Chem* **290**, 22423-22434.

de Farias Neto, A.L., Hartman, G.L., Pedersen, W.L., Li, S., Bollero, G.A. and Diers, B.W. (2006) Irrigation and inoculation treatments that increase the severity of soybean sudden death syndrome in the field. *Crop Sci.* **46**, 2547-2554.

de Souza Carrocini, G.C., Venancio, L.P. and Bonini-Domingos, C.R. (2015) Screening of Transcription Factors Involved in Fetal Hemoglobin Regulation Using Phylogenetic Footprinting. *Evol Bioinform Online* **11**, 239-244.

Fehr, W.R., Caviness, C.E., Burmood, D.T. and Pennington, J.S. (1971) Stage of Development Descriptions for Soybeans, Glycine Max (L.) Merrill1. *Crop Sci.* **11**, 929-931.

Fosgerau, K. and Hoffmann, T. (2015) Peptide therapeutics: current status and future directions. *Drug Discov Today* **20**, 122-128.

Gibson, P., Shenaut, M., Njiti, V., Suttner, R. and Myers Jr, O. (1994) Soybean varietal response to sudden death syndrome. In: *Proc 24th Soybean Seed Res Conf, Chicago, Illinois* pp. 6-7.

Hackenberg, D., Wu, Y., Voigt, A., Adams, R., Schramm, P. and Grimm, B. (2012) Studies on differential nuclear translocation mechanism and assembly of the three subunits of the Arabidopsis thaliana transcription factor NF-Y. *Mol Plant* **5**, 876-888.

Hewezi, T., Howe, P., Maier, T.R., Hussey, R.S., Mitchum, M.G., Davis, E.L. and Baum, T.J. (2008) Cellulose binding protein from the parasitic nematode Heterodera schachtii interacts with Arabidopsis pectin methylesterase: cooperative cell wall modification during parasitism. *Plant Cell* **20**, 3080-3093.

Hillwig, M.S., Chiozza, M., Casteel, C.L., Lau, S.T., Hohenstein, J., Hernandez, E., Jander, G. and MacIntosh, G.C. (2016) Abscisic acid deficiency increases defence responses against Myzus persicae in Arabidopsis. *Mol Plant Pathol* **17**, 225-235.

Jones, D.C., Zheng, W., Huang, S., Du, C., Zhao, X., Yennamalli, R.M., Sen, T.Z., Nettleton, D., Wurtele, E.S. and Li, L. (2016) A Clade-Specific Arabidopsis Gene Connects Primary Metabolism and Senescence. *Front Plant Sci* **7**, 983.

Jones, D.T. (1999) Protein secondary structure prediction based on position-specific scoring matrices. *J Mol Biol* **292**, 195-202.

Kandel, Y.R., Bradley, C.A., Wise, K.A., Chilvers, M.I., Tenuta, A.U., Davis, V.M., Esker, P.D., Smith, D.L., Licht, M.A. and Mueller, D.S. (2015) Effect of Glyphosate Application on Sudden Death Syndrome of Glyphosate-Resistant Soybean Under Field Conditions. *Plant Disease* **99**, 347-354.

Katagiri, F., Thilmony, R. and He, S.Y. (2002) The Arabidopsis thaliana-pseudomonas syringae interaction. *The Arabidopsis book / American Society of Plant Biologists* **1**, e0039.

Kato, T., Shimono, Y., Hasegawa, M., Jijiwa, M., Enomoto, A., Asai, N., Murakumo, Y. and Takahashi, M. (2009) Characterization of the HDAC1 complex that regulates the sensitivity of cancer cells to oxidative stress. *Cancer Res* **69**, 3597-3604.

Li, L., Foster, C.M., Gan, Q., Nettleton, D., James, M.G., Myers, A.M. and Wurtele, E.S. (2009a) Identification of the novel protein QQS as a component of the starch metabolic network in Arabidopsis leaves. *Plant Journal* **58**, 485-498.

Li, L. and Wurtele, E.S. (2015) The QQS orphan gene of Arabidopsis modulates carbon and nitrogen allocation in soybean. *Plant biotechnology journal* **13**, 177-187.

Li, L., Zheng, W., Zhu, Y., Ye, H., Tang, B., Arendsee, Z.W., Jones, D., Li, R., Ortiz, D., Zhao, X., Du, C., Nettleton, D., Scott, M.P., Salas-Fernandez, M.G., Yin, Y. and Wurtele, E.S. (2015) QQS orphan gene regulates carbon and nitrogen partitioning across species via NF-YC interactions. *Proc Natl Acad Sci U S A* **112**, 14734-14739.

Li, S., Hartman, G. and Chen, Y. (2009b) Evaluation of aggressiveness of Fusarium virguliforme isolates that cause soybean sudden death syndrome. *Journal of Plant Pathology* **91**, 77-86.

Moeinvaziri, F. and Shahhoseini, M. (2015) Epigenetic role of CCAAT box-binding transcription factor NF-Y on ID gene family in human embryonic carcinoma cells. *IUBMB Life* **67**, 880-887.

Nardini, M., Gnesutta, N., Donati, G., Gatta, R., Forni, C., Fossati, A., Vonrhein, C., Moras, D., Romier, C., Bolognesi, M. and Mantovani, R. (2013) Sequence-specific transcription factor NF-Y displays histone-like DNA binding and H2B-like ubiquitination. *Cell* **152**, 132-143.

Niblack, T., Arelli, P., Noel, G., Opperman, C., Orf, J., Schmitt, D., Shannon, J. and Tylka, G. (2002) A revised classification scheme for genetically diverse populations of Heterodera glycines. *Journal of Nematology* **34**, 279.

Niblack, T., Tylka, G.L., Arelli, P., Bond, J., Diers, B., Donald, P., Faghihi, J., Ferris, V., Gallo, K. and Heinz, R.D. (2009) A standard greenhouse method for assessing soybean cyst nematode resistance in soybean: SCE08 (standardized cyst evaluation 2008). *Plant Health Progress* **10**.

Ouali, M. and King, R.D. (2000) Cascaded multiple classifiers for secondary structure prediction. *Protein Sci* **9**, 1162-1176.

Ripodas, C., Clua, J., Battaglia, M., Baudin, M., Niebel, A., Zanetti, M.E. and Blanco, F. (2014) Transcriptional regulators of legume-rhizobia symbiosis: nuclear factors Ys and GRAS are two for tango. *Plant Signal Behav* **9**, e28847.

Romier, C., Cocchiarella, F., Mantovani, R. and Moras, D. (2003) The NF-YB/NF-YC structure gives insight into DNA binding and transcription regulation by CCAAT factor NF-Y. *J Biol Chem* **278**, 1336-1345.

Rost, B. (2001) Review: protein secondary structure prediction continues to rise. *J Struct Biol* **134**, 204-218.

Thilmony, R., Underwood, W. and He, S.Y. (2006) Genome-wide transcriptional analysis of the Arabidopsis thaliana interaction with the plant pathogen Pseudomonas syringae pv. tomato DC3000 and the human pathogen Escherichia coli O157:H7. *Plant J* **46**, 34-53.

Tina, K.G., Bhadra, R. and Srinivasan, N. (2007) PIC: Protein Interactions Calculator. *Nucleic acids research* **35**, W473-476.

Yang, C., Guo, R., Jie, F., Nettleton, D., Peng, J., Carr, T., Yeakley, J.M., Fan, J.-B. and Whitham, S.A. (2007) Spatial Analysis of Arabidopsis thaliana Gene Expression in Response to Turnip mosaic virus Infection. *Molecular Plant-Microbe Interactions* **20**, 358-370.

Zhu, X., Wang, Y., Pi, W., Liu, H., Wickrema, A. and Tuan, D. (2012) NF-Y recruits both transcription activator and repressor to modulate tissue- and developmental stage-specific expression of human gamma-globin gene. *PLoS One* **7**, e47175.
